# Noninvasive assessment of the lung inflammation-fibrosis axis by targeted imaging of CMKLR1

**DOI:** 10.1126/sciadv.adm9817

**Published:** 2024-06-19

**Authors:** Philip Z. Mannes, Taylor S. Adams, Samaneh Farsijani, Clayton E. Barnes, Joseph D. Latoche, Kathryn E. Day, Jessie R. Nedrow, Farida Ahangari, Naftali Kaminski, Janet S. Lee, Sina Tavakoli

**Affiliations:** ^1^ Department of Radiology, University of Pittsburgh, Pittsburgh, PA, USA.; ^2^ Medical Scientist Training Program, University of Pittsburgh, Pittsburgh, PA, USA.; ^3^ Section of Pulmonary, Critical Care and Sleep Medicine, Yale University School of Medicine, New Haven, CT, USA.; ^4^ Department of Epidemiology, University of Pittsburgh, Pittsburgh, PA, USA.; ^5^ Center for Aging and Population Health, University of Pittsburgh, Pittsburgh, PA, USA.; ^6^ Department of Medicine, University of Pittsburgh, Pittsburgh, PA, USA.; ^7^ Division of Pulmonary and Critical Care Medicine, Department of Medicine, Washington University, St. Louis, MO, USA.; ^8^ Heart, Lung, Blood, and Vascular Medicine Institute, University of Pittsburgh Medical Center, Pittsburgh, PA, USA.

## Abstract

Precision management of fibrotic lung diseases is challenging due to their diverse clinical trajectories and lack of reliable biomarkers for risk stratification and therapeutic monitoring. Here, we validated the accuracy of CMKLR1 as an imaging biomarker of the lung inflammation-fibrosis axis. By analyzing single-cell RNA sequencing datasets, we demonstrated *CMKLR1* expression as a transient signature of monocyte-derived macrophages (MDMφ) enriched in patients with idiopathic pulmonary fibrosis (IPF). Consistently, we identified MDMφ as the major driver of the uptake of CMKLR1-targeting peptides in a murine model of bleomycin-induced lung fibrosis. Furthermore, CMKLR1-targeted positron emission tomography in the murine model enabled quantification and spatial mapping of inflamed lung regions infiltrated by CMKLR1-expressing macrophages and emerged as a robust predictor of subsequent lung fibrosis. Last, high *CMKLR1* expression by bronchoalveolar lavage cells identified an inflammatory endotype of IPF with poor survival. Our investigation supports the potential of CMKLR1 as an imaging biomarker for endotyping and risk stratification of fibrotic lung diseases.

## Teaser

CMKLR1 is an imaging biomarker of the inflammation-fibrosis axis by identifying a macrophage subset enriched in lung fibrosis.

## INTRODUCTION

Pulmonary fibrosis is characterized by the replacement of the elastic and compliant lung interstitium by a disorganized and collagen-rich extracellular matrix (ECM) as a result of various diseases, such as infections, immunological diseases, acute respiratory distress syndrome (ARDS), and radiation in addition to a diverse category of idiopathic interstitial lung diseases (ILDs) (*1*). This etiological diversity along with the variability of the clinical course and treatment response of patients has presented major challenges to the precision management of fibrotic lung diseases (*2*). While some patients experience nonprogressive disease or gradual physiological deterioration over years, others face progressive fibrosis, occasionally accompanied by unpredictable episodes of acute exacerbations, leading to rapid decompensation and, in some cases, death within months (*1*, *3*). Despite recent advances in antifibrotic and/or immunomodulatory treatments that can mitigate disease progression in some patients, current clinical and laboratory biomarkers are insufficient for assessing the risk of progressive disease, identifying patients who may respond more favorably to various therapeutic interventions, or monitoring therapeutic responses (*2*–*4*). Therefore, diagnostic strategies for noninvasive monitoring of pathological drivers of adverse ECM remodeling are vital for precision management of fibrotic lung diseases.

Dysregulated immune responses play a pivotal role in driving adverse ECM remodeling across various fibrotic lung diseases, which may be best exemplified by fibrosis caused by pneumonia, ARDS, and rheumatological diseases (*1*, *4*–*8*). Even in diseases with less robust inflammatory profiles, such as idiopathic pulmonary fibrosis (IPF), a growing body of evidence supports the role of dysregulated immune responses in the pathogenesis of lung fibrosis (*4*, *5*, *8*). The accumulation of monocyte-derived macrophages (MDMφ), which replace or outnumber tissue-resident alveolar macrophages, has recently emerged as a crucial factor in promoting lung fibrosis (*4*, *9*–*11*). Under the homeostatic state, tissue-resident alveolar macrophages effectively eliminate pathogenic stimuli without provoking an inflammatory response (*12*). However, MDMφ recruited during lung injuries continue to exhibit proinflammatory and profibrotic functions for extended periods, even after the initial triggering insult has resolved, and have emerged as key drivers of adverse ECM remodeling (*6*, *7*, *13*, *14*). Consequently, tracking the in vivo kinetics of MDMφ accumulation holds promise to assess the lung inflammation-fibrosis axis.

Positron emission tomography (PET) is a promising technology for monitoring dynamic changes in lung immune cell populations. For instance, the feasibility of detecting monocyte influx has been shown by targeted imaging of C-C chemokine receptor 2 (CCR2) in murine models of lung fibrosis and in a first-in-human study of patients with IPF (*15*). However, the long-term retention of MDMφ independent of ongoing monocyte influx may not be captured by CCR2-targeting due to the rapid down-regulated expression of CCR2 upon monocyte differentiation to macrophages (*16*–*18*). To address this gap, we recently described that targeted imaging of chemokine-like receptor 1 (CMKLR1), a marker primarily expressed by MDMφ but not by tissue-resident alveolar macrophages, enables monitoring of the total burden of lung MDMφ in a mouse model of acute lung injury (*16*). We also demonstrated that *CMKLR1*-expressing lung macrophages transcriptionally resemble a recently described subset of CD163^+^ and LGMN^+^ MDMφ with a profibrotic phenotype in patients with COVID-19 (*16*, *19*). In the present study, we investigated the potential of CMKLR1-targeted PET to monitor the kinetics of MDMφ accumulation in a murine model of fibrotic lung injury and to determine the clinical relevance of CMKLR1 as a biomarker for endotyping and prognostication of fibrotic lung diseases.

## RESULTS

### Combined analysis of two single-cell RNA sequencing datasets provides an updated landscape of macrophages in lung fibrosis

**Fig. 1.**
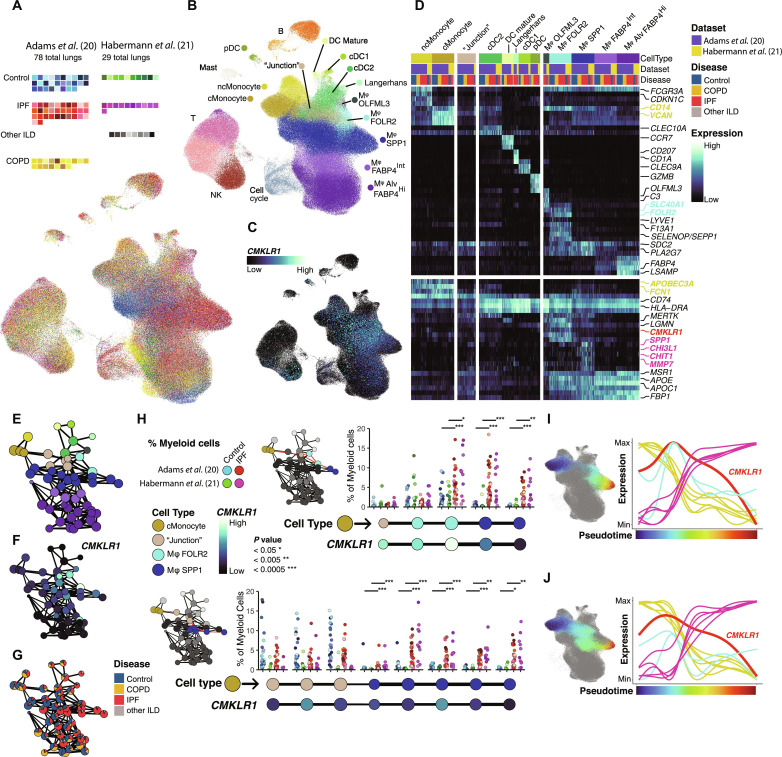
scRNA-seq analysis of human lungs in disease and health. (**A**) Waffle plots representing the number of subjects per disease state, per dataset (top) and UMAP of cells labeled by subject (bottom). (**B**) UMAP of cells labeled by cell type. (**C**) UMAP and (**D**) heatmap of normalized gene expression across cell types, datasets, and diseases. Each column represents the average gene expression for one subject per cell type. The upper section corresponds to sensitive and specific markers per cell type; the lower section corresponds to either broadly observed or disease-restricted features. Values are min-max normalized within each dataset. (**E**) Partition-based graph abstraction (PAGA) of subclustered myeloid cells in a layout corresponding to the earlier UMAP with vertices colored by their respective cell type. All edges have a confidence value >0.65. (**F**) PAGA with average normalized expression of *CMKLR1*. (**G**) PAGA with relative disease portion makeup. (**H**) IPF and control representation across two PAGA subgraph trajectories ordered from cMonocyte toward IPF-enriched macrophage archetype. The Mann-Whitney *U* test was used to compare the proportion of myeloid cells in each cluster between IPF and control lungs. Independent tests were applied to each dataset; *P < 0.05, **P <0.01, ***P < 0.001. (**I** to **J**) Generalized additive model (GAM) fits of gene expression as a function of diffusion pseudotime for each aforementioned trajectory. Line colors correspond to cMonocyte features (yellow), FOLR2 macrophage features (cyan), *CMKLR1* (red), and IPF features (magenta) with corresponding colors in above heatmap.

To investigate the role of CMKLR1 in IPF lung immune cells, we performed exploratory data analysis of two publicly available single-cell RNA sequencing (scRNA-seq) datasets of whole lungs with >300,000 immune cells: Adams *et al.* (*20*) includes 28 controls, 32 IPF, and 18 chronic obstructive pulmonary disease (COPD) lungs; Habermann *et al.* (*21*) consists of 10 control, 11 IPF, and eight assorted ILD lungs (Fig. 1, A to C). This combined sample size enabled a more granular perspective of macrophage subpopulations in human lung fibrosis than previously reported.

Macrophage phenotypes were first broadly classified into two main groups: alveolar and nonalveolar. Alveolar macrophages were defined by expression patterns of genes, including *FABP4*, *LSAMP*, and *PPARG* (Fig. 1D and data file S1), consistent with previous descriptions (*22*). We further subdivided these cells into Alv-Mφ (FABP4^Hi^) and FABP4^Int^-Mφ, where the latter had relatively lower expression of the same distinguishing features (fig. S1). Alv-Mφ and FABP4^Int^-Mφ were transcriptomically distinguishable phenotypes that co-occurred in every sample, irrespective of dataset or disease state (Fig. 1D) with sufficient differences in global gene expression patterns to separate these populations in the feature-space of the uniform manifold approximation and projection (UMAP) layout. However, the dynamic relationship between FABP4^Int^-Mφ and FABP4^Hi^-Alv-Mφ, such as a transitioning state between the two subsets or a stable subset observed in a transient state of activity, remains unknown.

The remaining group of nonalveolar macrophages was further divided into three groups. The largest and most indeterminate group was SPP1-Mφ, which had elevated *SPP1*, *PLA2G7*, and *SDC2*, but were otherwise classified by exclusion. IPF profibrotic macrophages were a pronounced archetype among SPP1**-**Mφ (*20*, *22*) (Fig. 1, A and D); a separate inflammatory archetype from a single control outlier also belonged to this SPP1-Mφ (*20*) (Fig. 1A).

Distinguishing features of FOLR2-Mφ include *LYVE1*, *F13A1*, *SEPP1*, *LILRB5*, and *CCL13*. Recent studies have brought attention to the ubiquity of FOLR2-Mφ throughout the body (*23*) and the role they play in the lung following injury (*24*). The role of FOLR2-Mφ has been overlooked in previous scRNA-seq studies of interstitial lung disease (*20*–*22*) and their status in IPF remains poorly characterized.

We also identified a rare, third population of nonalveolar macrophage–labeled OLFML3-Mφ. These cells were classified as macrophages based on the shared expression of *MERTK*, *PLA2G7*, and *C1Q* complex genes, though they also have dendritic features like *FCGR2B*, *FCER1A*, and elevated *MHC-II* (Fig. 1D and data file S1). In the context of other macrophages, these cells share the expression of *SLC40A1* and *OLFM2B* with FOLR2-Mφ but otherwise lack the expression of the remaining FOLR2-Mφ expression suite. Across samples in both datasets, OLMFL3-Mφ cells uniquely express *C3*, *FCGBP*, *PLD4*, *GPR34*, *IGSF21*, and *OLFML3* (Fig. 1D and data file S1). These cells were commonly found across IPF samples and only rarely found in controls (Fig. 1, A and D).

### *CMKLR1* expression is a hallmark of nascent MDMφ enriched in pulmonary fibrosis and gradually declines as macrophages acclimate to the fibrotic microenvironment

Between both datasets, *CMKLR1* expression was most frequently detected across macrophage populations (Fig. 1, C and D, and data file S2), consistent with our previous finding in patients with COVID-19 (*16*). Irrespective of the dataset or whether the cells were from IPF or control lungs, FOLR2-Mφ had the highest detection frequency and average normalized expression of *CMKLR1* across all immune cell types. The next highest ranked cell types in descending order were OLFML3-Mφ, SPP1-Mφ, FABP4^Int^-Mφ, and, lastly, FABP4^Hi^-Alv-Mφ (data file S2). These findings are consistent with our previous analyses where nonalveolar macrophages expressing *CMKLR1* in COVID-19 lungs also expressed FOLR2-Mφ features like *LYVE1* and *F13A1* (*16*) (Fig. 1D) and how *CMKLR1* expression was observed highest among *FOLR2*^+^ and *SPP1*^+^ interstitial macrophage in patients with cystic fibrosis (*16*).

An exploratory analysis of *CMKLR1* expression in different diseases suggested a preferential expansion of *CMKLR1*-expressing macrophages in IPF compared to other ILD entities (i.e., chronic hypersensitivity pneumonitis, nonspecific interstitial pneumonia, unclassifiable ILD, and sarcoidosis) and COPD (fig. S2). However, considering the relatively lower representation of lung cells from these disease states compared to IPF, further studies are required to characterize disease-specific patterns of *CMKLR1* expression in various ILDs and in different endotypes or stages of COPD.

Changes in *CMKLR1* expression were not commensurate with the boundaries of nonalveolar macrophage cell types: we observed more *CMKLR1* variation among SPP1-Mφ than between SPP1-Mφ and adjacent macrophages (Fig. 1, B and C). Summarizing expression values across cell types in this context has limitations. Qualitatively, we observed *CMKLR1* expression peaking across a variety of nonalveolar macrophages near their boundaries with the monocyte/cDC2/macrophage intermediate population “junction.” Expression gradually declined in a diffuse pattern where ultimately the most extremal cell types and cell states have low-to-no detectable *CMKLR1* (Fig. 1C). This pattern indicates that *CMKLR1* expression is broadly associated with nascent MDMφ.

Considering that IPF severity is typically worse in the lung bases, we compared *CMKLR1* expression by immune cells between the upper and lowers lobes of patients with IPF versus control participants using the scRNA-seq dataset from Morse *et al.* (*25*) [Gene Expression Omnibus (GEO) accession GSE128033]. Consistent with our results from the Adams *et al.* (*20*) and Habermann *et al.* (*21*) datasets, *CMKLR1* expression was highest in SPP1-Mφ and FOLR2-Mφ with a low expression in Alv-Mφ and extremal profibrotic SPP1-Mφ (i.e., SPP1^+^ macrophages with the highest intensity of the profibrotic gene expression) (fig. S3). Upper lobes from patients with IPF had more FABP4^+^ alveolar macrophages and fewer IPF-associated profibrotic SPP1^+^ macrophages than the lower lobes but similarly elevated numbers of *CMKLR1*^+^ macrophages (IPF upper lobe: 8.4% and lower lobe: 8.9% versus control upper lobe: 2.3% and lower lobe: 0.8%). While this requires further investigation, it may be speculated that accumulation of *CMKLR1*^+^ macrophages in the lungs may represent a more reliable biomarker of early disease than the SPP1^+^ profibrotic macrophages described in end-stage disease.

To prepare for trajectory-based analysis, we assessed the robustness of cell-cell relationships to ensure that downstream pseudotime orderings would be constrained to plausible outcomes. Monocytes, macrophages, and dendritic cells contiguous in UMAP space (Fig. 1B) were isolated and reclustered with a high-resolution parameter yielding over 50 granular subclusters organized below the level of cell types. Cell-cell connections between these subpopulations were statistically modeled with partition-based graph abstraction (PAGA) (*26*) and intercluster connections with high confidence values were preserved for downstream analysis (Fig. 1E).

Plotting a trajectory from monocytes toward extremal macrophage fates, we observed a branching event between FOLR2-Mφ and SPP1-Mφ that occurred among junction clusters (Fig. 1E), consistent with how differentiation occurs in adult mouse lungs (*24*), in which *Folr2*^+^ interstitial macrophages are gradually replaced by circulating monocytes over time. Although under healthy conditions *Folr2*^+^ interstitial macrophages have a longer half-life than other interstitial macrophages in adult mice (*24*, *27*, *28*), their rate of turnover under fibrotic conditions is unknown. Unexpectedly, one FOLR2-Mφ subcluster featuring the highest overall *CMKLR1* expression (Fig. 1F) was overrepresented in IPF samples (Fig. 1G) and had several high confidence connections directly to subclusters of the SPP1-Mφ IPF profibrotic archetype (Fig. 1E). These patterns suggest that after recruited lung monocytes diverge toward either SPP1-Mφ or FOLR2-Mφ states, IPF macrophages from either population may converge into a common profibrotic SPP1-Mφ archetype, although this should be interpreted within the constraints of pseudotime analysis, which cannot distinguish bona fide phenotypic transitions from phenotypic similarities.

We then focused on two putative trajectories that both start with classical monocytes (cMonocytes) and end with profibrotic SPP1-Mφ, one via FOLR2-Mφ and the other via SPP1-Mφ (Fig. 1H). Following the FOLR2-Mφ PAGA trajectory, normalized *CMKLR1* expression peaked in a subcluster of FOLR2-Mφ, which was overrepresented across IPF lungs in both datasets (Fig. 1H). Generalized additive models (GAMs) that fit the corresponding cell-level pseudotime trajectory showed that *CMKLR1* expression is rising concurrently with the decline of monocyte features, peaking simultaneously with *FOLR2* as profibrotic gene expression initializes, then gradually declining as *FOLR2* is lost and profibrotic features gradually rise (Fig. 1I). Similarly, the trajectory restricted to SPP1-Mφ had *CMKLR1* expression peaking while monocyte features start declining and expression of profibrotic features begin to rise (Fig. 1J). *CMKLR1* was represented in these models as a transient hallmark of MDMφ among both FOLR2-Mφ and SPP1-Mφ states, and that *CMKLR1* expression gradually declines as macrophages acclimated to the fibrotic microenvironment.

### MDMφ are the major driver of the uptake of CMKLR1-targeting peptides in a murine model of fibrotic lung injury

We recently reported the feasibility of targeted imaging of CMKLR1 in acute lung injury using a proteolytically stable and high-affinity copper-64–labeled radiotracer ([^64^Cu]NODAGA-CG34) (*16*) derived from an analog of the C-terminus of chemerin (*29*). To leverage the advantage of flow cytometry for quantifying the abundance of various CMKLR1-expressing immune cells and their respective contribution to radiotracer uptake during different stages of fibrotic lung injury, we synthesized fluorochrome-labeled analogs of [^64^Cu]NODAGA-CG34 by replacing the chelator moiety (NODAGA) of the tracer with fluorescein (6CF-CG34) or TAMRA (6TAM-CG34) (fig. S4). We confirmed that the fluorochrome-modified CG34 peptides exhibited similar pharmacodynamic properties as NODAGA-CG34, including comparable binding/uptake by mouse peritoneal macrophages, which constitutively express CMKLR1 (*16*) and Chinese hamster ovary (CHO) cells transiently transfected with murine CMKLR1 (mCMKLR1), as well as displacement by NODAGA-CG34 in competitive uptake assays in peritoneal macrophages (fig. S5, A to H). Furthermore, we established that CG34-derived peptides are internalized in a CMKLR1-selective manner (fig. S5I) with no significant binding/uptake by the two related G protein–coupled receptors of chemerin, namely, chemokine (C-C motif) receptor–like 2 (CCRL2) and G protein–coupled receptor 1 (GPR1) (fig. S5, F and G). We also demonstrated the high affinity of fluorochrome-conjugated CG34 to human CMKLR1 (fig. S5H), using transiently transfected CHO cells, which was comparable to its affinity to mouse CMKLR1.

**Fig. 2.**
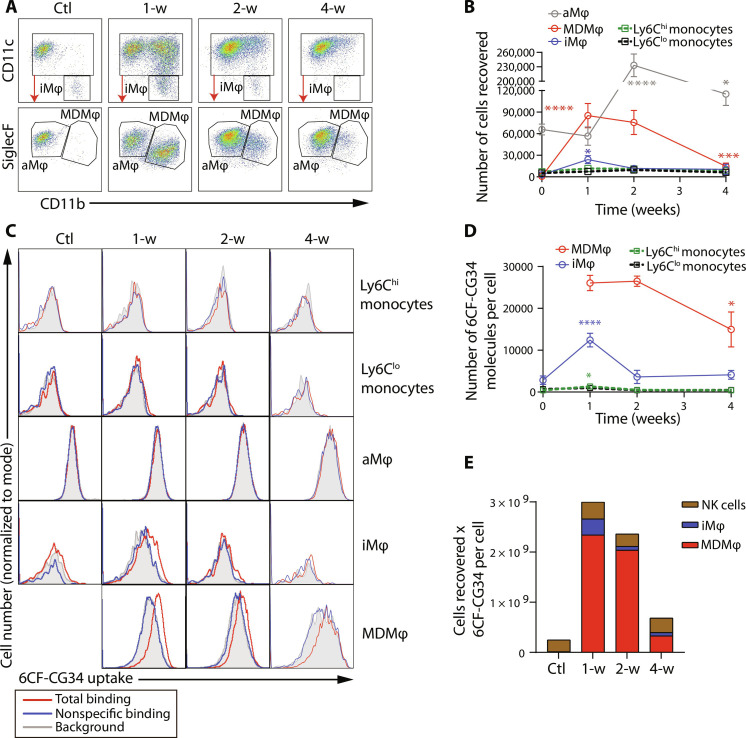
Flow cytometric identification of cells contributing to the uptake of CMKLR1-targeting peptide. (**A**) Alveolar macrophages, interstitial macrophages, and MDMφ were identified based on differential expressions of CD11b, CD11c, and SiglecF. (**B**) Kinetic changes in the absolute number of recovered monocyte and macrophage subsets revealed a marked infiltration of the lungs during the first 2 weeks after bleomycin administration, partially resolved by week 4. (**C**) Representative histograms reveal the uptake of CMKLR1-targeting fluorescent ligand 6CF-CG34 by various lung monocyte/macrophage populations in bleomycin- or PBS-treated mice [gray: background/autofluorescence; red: total binding of 6CF-CG34 in the absence of Chem_145–157_ (a high-affinity CMKLR1 ligand derived from the amino acids 145 to 157 of chemerin used as a competitive blocker of 6CF-CG34 uptake); blue: nonspecific binding of 6CF-CG34 in the presence of 10 μM Chem_145–157_]. (**D**) Quantification of specific (total minus nonspecific) uptake of 6CF-CG34 by selected monocyte and macrophage populations. MDMφ demonstrated the highest 6CF-CG34 uptake at 1 and 2 weeks postbleomycin, while interstitial macrophages demonstrated a transient increase in 6CF-CG34 uptake at 1 week postbleomycin. A stacked-bar chart summary of cell counts for CMKLR1 expressing populations multiplied by the cellular uptake of 6CF-CG34 (molecules per cell) (**E**) showed that MDMφ accounted for most of the lung uptake of 6CF-CG34 at 1 and 2 weeks following bleomycin. *n* = 3 male and 3 female mice for control and 1- and 2-week postbleomycin groups; *n* = 4 male and 4 female mice for the 4-week group. aMφ, alveolar macrophages; iMφ, interstitial macrophages; NK cells, natural killer cells. Comparisons were made between various treatment time points to the control group for alveolar macrophages, interstitial macrophages, Ly6C^hi^ monocytes, and Ly6C^lo^ monocytes or to 1-week treatment group for MDMφ (as the control group is omitted). *P < 0.05, **P < 0.01, ***P < 0.001, and ****P < 0.0001. Statistical significance was calculated using a one-way ANOVA with a post-hoc two-tailed Fisher’s exact test.

Using the gating strategy outlined in fig. S6, we identified and quantified major lung immune cell populations in control mice and at different time points (1, 2, and 4 weeks) after intratracheal bleomycin administration (fig. S7). These time points approximately correspond to the peak pneumonitis phase, early ECM remodeling with continued pneumonitis, and the resolution of inflammation with established fibrosis, respectively (*30*). We were particularly interested in characterizing different macrophage subsets as our recent work revealed that increased uptake of [^64^Cu]NODAGA-CG34 in a preclinical model of acute lung injury was primarily driven by MDMφ (*16*). To this end, we used a previously established flow cytometric method to distinguish MDMφ (CD11b^hi^/CD11c^hi^/SiglecF^int^) from tissue-resident alveolar macrophages (CD11b^lo^/CD11c^hi^/SiglecF^hi^), as validated by lineage tracing studies (*6*). At baseline, tissue-resident alveolar macrophages were the predominant population recovered from the lungs with fewer interstitial macrophages and negligible MDMφ (Fig. 2, A and B). It is noteworthy that the low abundance of interstitial macrophages at baseline is a reflection of the lower recovery of these cells compared to alveolar macrophages during single-cell preparation and the number of interstitial macrophages is much higher in lung parenchyma as demonstrated by stereological studies compared to flow cytometry (*31*). At 1 week following bleomycin, a significant accumulation of MDMφ was observed, whereas the number of alveolar macrophages remained stable (Fig. 2, A and B). By the second week, the number of MDMφ remained similar to that at 1 week, but there was a substantial increase in the number of alveolar macrophages. MDMφ at 2 weeks postbleomycin showed increasing resemblance to tissue-resident alveolar macrophages (i.e., increased SiglecF and decreased CD11b expression) (Fig. 2A), indicating their progressive differentiation toward the alveolar macrophage phenotype. By the fourth week, the number of both MDMφ and alveolar macrophages returned to baseline levels. In contrast, the number of lung monocytes (Ly6C^hi^ and Ly6C^lo^) remained relatively stable from week 1 to week 4 after bleomycin-induced injury.

We observed a high uptake of 6CF-CG34 by MDMφ during the initial 2 weeks following bleomycin-induced injury, which trended toward a decrease by the fourth week (Fig. 2, C and D). Interstitial macrophages displayed only a transient increase in 6CF-CG34 uptake at 1 week, which returned to the baseline level at 2 and 4 weeks postbleomycin. However, alveolar macrophages and monocytes (Ly6C^hi^ and Ly6C^lo^) did not exhibit an appreciable specific uptake of 6CF-CG34 throughout the 4-week period following injury. The only other immune cell type that exhibited significant 6CF-CG34 uptake was natural killer cells, but their abundance or CMKLR1 expression remained unchanged after bleomycin-induced injury (fig. S7).

To estimate the relative contribution of different immune cell populations to the total uptake of the fluorescent CMKLR1-selective ligand, we multiplied the cell count of the three major CMKLR1-expressing cell types (MDMφ, interstitial macrophages, and natural killer cells) by the number of 6CF-CG34 molecules taken up per cell (Fig. 2E). At baseline, we observed low 6CF-CG34 uptake. However, at 1 and 2 weeks postinjury, there was a significant increase in total 6CF-CG34 uptake, primarily driven by the accumulation of MDMφ, which accounted for 78 and 86% of the observed uptake by monocyte/macrophages and natural killer cells at 1 and 2 weeks, respectively. By 4 weeks, the 6CF-CG34 uptake had decreased to approximately baseline levels, reflecting a reduction in the abundance of MDMφ and their CMKLR1 expression level. Natural killer cells made a minimal contribution to the total 6CF-CG34 uptake in bleomycin-injured lungs, constituting only 11% at 1 and 2 weeks compared to 95% at baseline, due to their markedly lower abundance compared to MDMφ in injured lungs.

### [^64^Cu]NODAGA-CG34 PET monitors lung inflammation throughout the progression of bleomycin-induced fibrotic injury

**Fig. 3.**
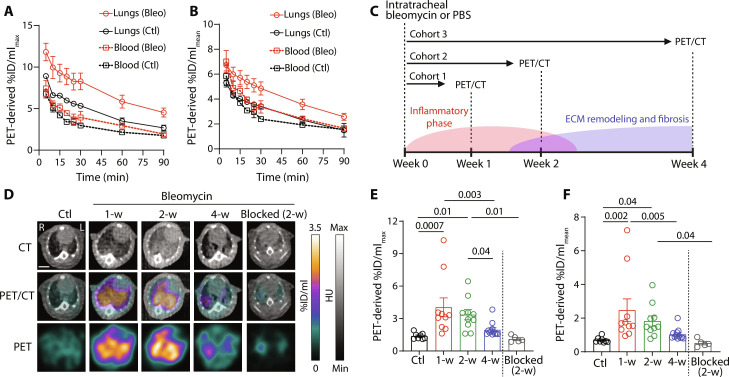
[^64^Cu]NODAGA-CG34 PET/CT in a murine model of bleomycin-induced lung fibrosis. (**A** and **B**) Kinetic [^64^Cu]NODAGA-CG34 uptake in the lungs and blood pool activity, quantified as the maximal % injected dose (ID)/ml_max_ (A) and mean %ID/ml_mean_ (B), over the first 90 min following radiotracer injection to bleomycin-treated (week 1, *n* = 3) and control (*n* = 2) mice. At 90 min, there was a high accumulation of [^64^Cu]NODAGA-CG34 in the lungs of bleomycin-treated versus control mice, while the radiotracer was mostly cleared from the circulation. (**C**) The design of the time-course study for quantifying [^64^Cu]NODAGA-CG34 uptake at distinct stages of bleomycin-induced fibrotic lung injury. (**D**) Representative axial CT, PET, and coregistered PET/CT images acquired ~90 min following administration of [^64^Cu]NODAGA-CG34 to a control mouse or mice at 1, 2, or 4 weeks postbleomycin injury (scale bar, 5 mm). Diffuse lung uptake of [^64^Cu]NODAGA-CG34 was observed in the lungs of mice at 1 and 2 weeks postbleomycin, which returned to near baseline by 4 weeks. The specificity of radiotracer uptake was confirmed by blockade of [^64^Cu]NODAGA-CG34 uptake in the lungs when co-injected with ~100-fold molar excess of nonradiolabeled NODAGA-CG34. PET-derived quantification demonstrated significant increases in the lung uptake of [^64^Cu]NODAGA-CG34 both measured as %ID/ml_max_ (**E**) and %ID/ml_mean_ (**F**) at 1 and 2 weeks postbleomycin. %ID/ml_max_ and %ID/ml_mean_ data in (A), (B), (E), and (F) represent the average values of the left and right lungs for each mouse. Ctl, control; Bleo, bleomycin. Statistical significance between groups was calculated using a one-way ANOVA with a two-tailed Fisher’s exact post hoc test.

Building on our flow cytometry findings, we investigated the potential of CMKLR1-targeted PET using [^64^Cu]NODAGA-CG34 to noninvasively track the kinetics of MDMφ accumulation in bleomycin-induced lung injury. Kinetic PET studies in 1 week postbleomycin versus control mice revealed substantial blood pool radioactivity during the initial 30 min after tracer administration hindering visualization and accurate quantification of [^64^Cu]NODAGA-CG34 uptake in the lungs (Fig. 3, A and B, and fig. S8). There was a progressive clearance of blood pool activity from 30 to 60 min, reaching a near plateau from 60 to 90 min. Therefore, for the remainder of the study, radiotracer quantifications were performed on static scans ~90 min post-tracer.

In line with flow cytometric results, visual examination of PET demonstrated increased lung uptake of [^64^Cu]NODAGA-CG34 at 1 and 2 weeks following bleomycin, which decreased to levels nearly similar to those of control mice by 4 weeks (Fig. 3, C and D). Quantitative analysis of PET corroborated our qualitative assessment by demonstrating significant increases in the lung uptake of [^64^Cu]NODAGA-CG34 at 1 and 2 weeks postbleomycin both in terms of the uptake in the maximally affected lung regions [i.e., maximal percent injected dose per ml (%ID/ml_max_); Fig. 3E] and the average global uptake throughout the lungs [i.e., mean percent injected dose per ml (%ID/ml_mean_); Fig. 3F]. We confirmed the specificity of radiotracer uptake by demonstrating the blockade of the lung uptake of [^64^Cu]NODAGA-CG34 upon co-injection of excess nonradiolabeled NODAGA-CG34 (Fig. 3, D and F).

**Fig. 4.**
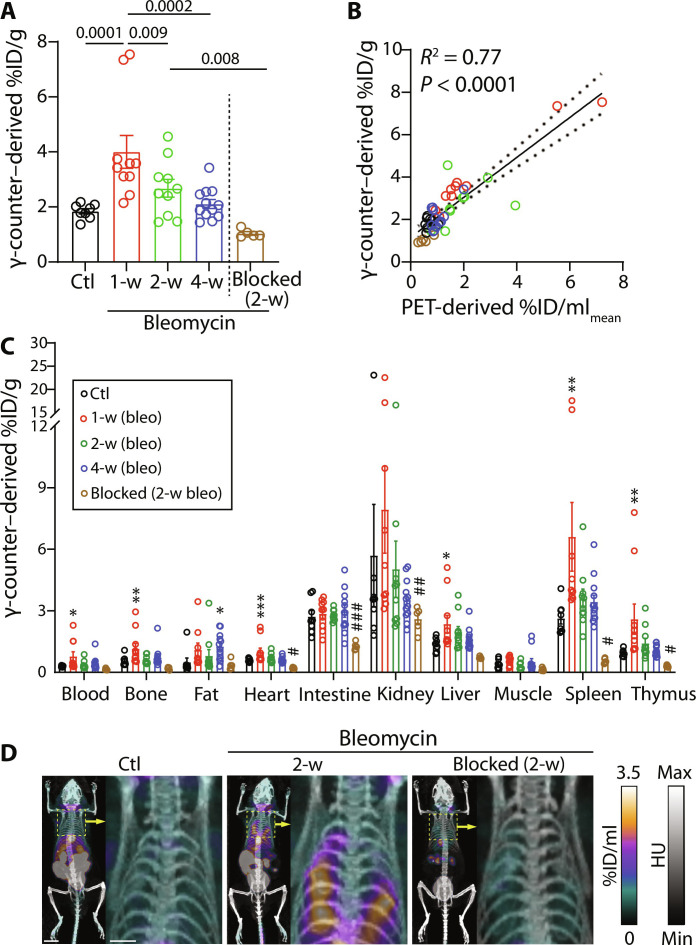
Ex vivo measurement of [^64^Cu]NODAGA-CG34 uptake and biodistribution. (**A**) Ex vivo γ-counting confirmed significantly increased lung uptake of [^64^Cu]NODAGA-CG34 at 1 and 2 weeks postbleomycin, which returned to baseline values at week 4. (**B**) The strong correlation between in vivo PET-derived and ex vivo γ-counting–derived measurements of [^64^Cu]NODAGA-CG34 uptake in the lungs confirms the accuracy of PET for noninvasive quantification of tracer uptake. (**C**) Biodistribution of [^64^Cu]NODAGA-CG34 was determined by γ-counting–derived of harvested organs (* or # *P* < 0.05; ** or ## *P* < 0.01; *** or ### *P* < 0.001; * represents comparison between control and nonblocked groups; # represents comparison between 2-week and blocked groups). (**D**) Representative full body PET/CT maximal intensity projections (scale bars, 9 mm) and magnified views of the thorax (yellow boxes, scale bars, 4 mm) to visualize [^64^Cu]NODAGA-CG34 uptake in the lung and other organs. Statistical significance between groups in (A) and (C) was calculated using a one-way ANOVA with a two-tailed Fisher’s exact post hoc test.

Quantification of radiotracer uptake using ex vivo γ-counting, as the gold standard method, yielded similar results with significant increases in radiotracer uptake at 1 and 2 weeks postbleomycin (Fig. 4A). There was also a strong correlation between in vivo PET-derived and ex vivo γ-counting–derived measurements, which establishes the accuracy of PET-derived quantification of [^64^Cu]NODAGA-CG34 uptake in the lungs (*R*^2^ = 0.77 and *P* < 0.0001) (Fig. 4B). In addition to the lungs, significant specific uptake of [^64^Cu]NODAGA-CG34 was present in several CMKLR1-rich organs, including liver, spleen, and thymus (Fig. 4, C and D).

### [^64^Cu]NODAGA-CG34 PET delineates inflamed lung regions infiltrated by CMKLR1-expressing macrophages

**Fig. 5.**
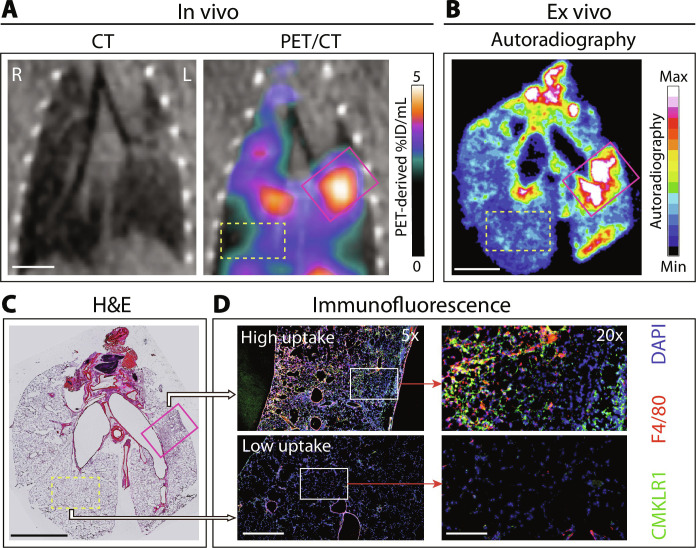
Colocalization of [^64^Cu]NODAGA-CG34 uptake with inflamed lung regions infiltrated by CMKLR1-expressing macrophages. Representative PET/CT images (**A**) from a bleomycin-treated (1 week) mouse reformatted to the plane of ex vivo autoradiography (**B**) and hematoxylin and eosin (H&E)–stained (**C**) and immunostained tissues (**D**) demonstrate colocalization of [^64^Cu]NODAGA-CG34 uptake with inflamed lung regions densely infiltrated by CMKLR1-expressing F4/80^+^ macrophages (pink box). Regions of low [^64^Cu]NODAGA-CG34 uptake (yellow box) demonstrate minimal expression of CMKLR1. Scale bars in CT, PET/CT, and autoradiography images: 4 mm; in H&E staining: 5-mm; in 5× immunofluorescence images: 800 μm; and in 20× immunofluorescence images: 200 μm. *n* = 3 male mice.

To confirm the accuracy of [^64^Cu]NODAGA-CG34 PET to provide a spatial map of inflamed lung tissues infiltrated by CMKLR1-expressing macrophages, we performed autoradiography of the lungs after completion of PET and conducted subsequent immunofluorescence staining for CMKLR1 and the macrophage marker F4/80 (Fig. 5). There was a robust colocalization between tracer uptake as detected by in vivo PET and the corresponding ex vivo autoradiography (Fig. 5, A and B), confirming the accuracy of noninvasive spatial localization of tracer uptake. The regions exhibiting high [^64^Cu]NODAGA-CG34 uptake corresponded to inflamed areas with an abundance of CMKLR1-expressing F4/80^+^ macrophages (Fig. 5, C and D). These results collectively indicate that [^64^Cu]NODAGA-CG34 PET enables quantitative monitoring and spatial mapping of MDMφ recruitment at various stages of bleomycin-induced fibrotic lung injury.

### [^64^Cu]NODAGA-CG34 PET predicts progressive fibrotic lung injury in the murine model

**Fig. 6.**
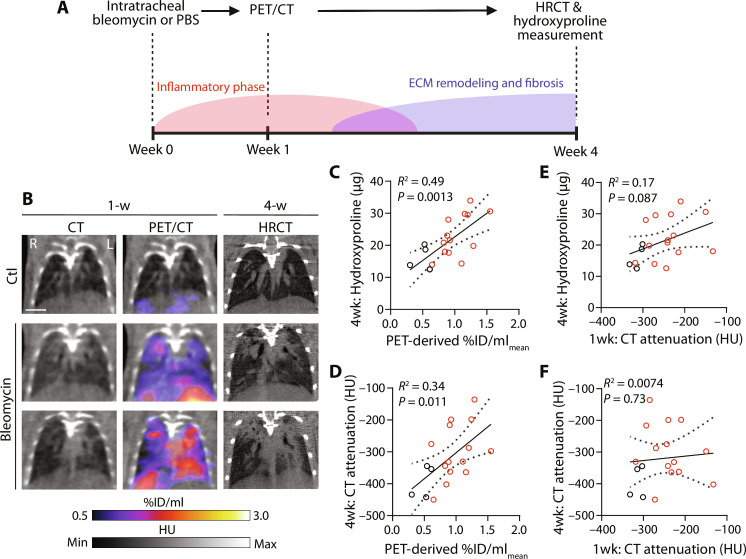
Prognostication of fibrotic lung injury by [^64^Cu]NODAGA-CG34 PET during the early stage of bleomycin-induced injury. (**A**) Experimental design to determine the potential of [^64^Cu]NODAGA-CG34 PET during the inflammatory phase of bleomycin-induced injury in predicting the severity of future lung fibrosis. (**B**) Representative PET/CT images (scale bar, 5 mm) demonstrate minimal lung uptake in a control mouse at 1 week following intratracheal PBS administration (top row) and no CT evidence of fibrosis at week 4. Examples from bleomycin-treated mice with low (middle row) versus high (lower row) tracer uptake demonstrate that [^64^Cu]NODAGA-CG34 uptake at week 1 colocalizes and subjectively correlates with the severity of future fibrosis at week 4 as visualized by high-resolution CT (HRCT). The severity of pulmonary fibrosis at 4 weeks, measured by the hydroxyproline contents (**C**) or increased lung attenuation (**D**), correlated with [^64^Cu]NODAGA-CG34 uptake in the corresponding lungs as measured by %ID/ml_mean_ at 1 week (Pearson correlation along with 95% confidence intervals). However, the increased lung attenuation at week 1 postbleomycin only trended toward a weak correlation with the hydroxyproline content at week 4 (**E**) and did not significantly correlate with the increased lung attenuation (**F**).

To determine the potential of CMKLR1-targeted PET to predict the progression of pulmonary fibrosis, we conducted a longitudinal study in which mice underwent PET/CT at week 1 postbleomycin and were followed until week 4 to assess the extent of fibrosis (Fig. 6A). As shown by representative images in Fig. 6B, regions of lung with high [^64^Cu]NODAGA-CG34 uptake at week 1 postbleomycin corresponded to regions that subsequently developed severe fibrosis as detected by high-resolution computed tomography (CT) conducted at week 4. Moreover, there was a significant correlation between PET-derived [^64^Cu]NODAGA-CG34 uptake at week 1 and the extent of lung fibrosis at week 4, as determined by their hydroxyproline content (Fig. 6C) and increased lung attenuation (Fig. 6D). In contrast, the severity of airspace opacification as measured by increased lung attenuation at week 1 postbleomycin only trended toward a weak correlation with the hydroxyproline content at week 4 (*R*^2^ = 0.17 and *P* = 0.087; Fig. 6E) and did not significantly correlate with the increased lung attenuation (Fig. 6F). Together, these results confirm the accuracy of [^64^Cu]NODAGA-CG34 uptake during the early stage of lung injury to predict the severity and distribution of future fibrosis, which complements the anatomical information obtained by CT.

### High *CMKLR1* expression by BAL cells identifies an inflammatory IPF endotype with a poor prognosis

**Fig. 7.**
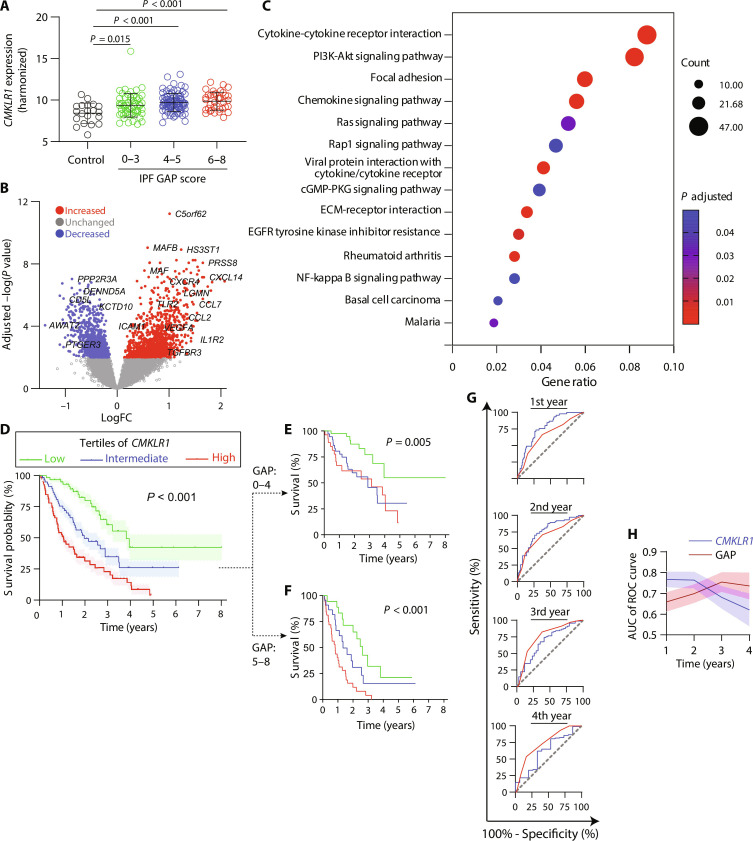
*CMKLR1* expression by BAL cells identifies an inflammatory and poor prognostic endotype of IPF. (**A**) *CMKLR1* expression by BAL cells is increased in patients with IPF compared to healthy controls but is not different across different categories of GAP scores. (**B**) Volcano plot of differentially expressed genes demonstrates numerous differentially expressed genes, including multiple markers of inflammation and ECM remodeling, in patients with high versus low *CMKLR1* expression. (**C**) Pathway enrichment analysis confirms significant enrichment of inflammatory and ECM remodeling pathways in patients with high versus low *CMKLR1* expression. (**D**) Kaplan-Meier survival curves based on *CMKLR1* expression alone (left) or after stratification by GAP score (**E** and **F**) demonstrates that high *CMKLR1* expression predicts poor survival. (**G** and **H**) ROC curves comparing *CMKLR1* expression versus GAP score for predicting patient mortality after indicated time points following BAL.

To explore the potential of CMKLR1 as a prognostic and endotyping biomarker, we conducted a secondary analysis of harmonized data (fig. S9) from a longitudinal cohort of IPF patients at three sites: Freiburg, Leuven, and Siena (*32*). We first compared *CMKLR1* expression in bronchoalveolar lavage (BAL) cells in healthy controls versus patients with IPF categorized based on their gender-age-physiology (GAP) index, a widely accepted clinical risk stratification model (*33*). We observed a higher *CMKLR1* expression in patients with IPF compared to controls, but no significant difference was present among patients in different GAP score categories (Fig. 7A). Notably, there was a marked heterogeneity in *CMKLR1* expression in patients with IPF independent of their GAP index.

To investigate whether the heterogeneity of *CMKLR1* expression may be leveraged to identify distinct inflammatory endotypes of IPF, we analyzed the BAL transcriptome of patients with *CMKLR1* expression above versus below the median value, which revealed numerous differentially expressed genes between the two groups (Fig. 7B and data file S3). Notably, patients with high *CMKLR1* expression had a significantly higher expression of many genes with established roles in inflammation, including chemokines/chemokine receptors (e.g., *CCL2*, *CCL7*, *CXCL14*, and *CXCR4*) and cytokines/cytokine receptors (e.g., *IL1R2* and *IL12RB2*), as well as ECM remodeling (e.g., *LGMN*, *MMP25*, *SPP1*, *COL1A1*, and *FGFBP2*) (Fig. 7B). Pathway enrichment analysis of the differentially overexpressed genes confirmed the involvement of inflammatory and ECM remodeling pathways in patients with high *CMKLR1* expression (Fig. 7C).

Next, we assessed the potential of *CMKLR1* expression as a prognostic biomarker by comparing the survival of patients in different tertiles of *CMKLR1* expression. Notably, higher *CMKLR1* expression was associated with a progressively worse prognosis with median survival of 388 days in the highest tertile versus 718 days in the middle and 1394 days in the lowest tertiles (Fig. 7D). Cox proportional hazards analysis adjusted for potential confounders confirmed that *CMKLR1* expression predicts mortality independent of age, sex, study site, and GAP score (fig. S10). We also explored the prognostic value of two additional biomarkers along the monocyte-to-macrophage differentiation spectrum, *CCR2*, as a monocytic marker and *SPP1*, as a hallmark of differentiated profibrotic macrophages. Intriguingly, high expression levels of both biomarkers were significantly associated with a worse survival (fig. S11). The divergence in survival curves for *SPP1* across the tertiles was overall similar to that of *CMKLR1* with a median survival of 432 days in the highest tertile compared to 958 days in the middle and 1404 days in the lowest tertiles. In contrast, the separation of the survival curves was less distinct for *CCR2* with the median survival of 586 days in the highest tertile versus 840 days in the middle and 1272 days in the lowest tertiles. These findings support that different biomarkers within the monocyte-to-macrophage differentiation spectrum offer valuable prognostic insights in IPF.

Furthermore, we confirmed the potential of *CMKLR1* expression to provide incremental risk stratification information beyond the existing clinical tools by demonstrating that *CMKLR1* expression was associated with a worse survival in analyses performed after stratification of patients to low GAP index (0 to 4; Fig. 7E) and high GAP index (5 to 8; Fig. 7F). We also performed receiver operating characteristic (ROC) analysis to compare the performance of *CMKLR1* expression and GAP index in predicting mortality risk at different time points after BAL (Fig. 7, G and H). The ROC analysis revealed that the *CMKLR1* expression had a higher area under the curve than the GAP score during the first 2 years, indicating its superior predictive ability over short-to-intermediate follow-up periods, whereas the GAP score was a better predictor of long-term (i.e., years 3 and 4) survival.

Together, these findings support that the heterogeneous *CMKLR1* expression may be harnessed to identify a distinct endotype of IPF with a more intense inflammatory and ECM remodeling profile associated with a high risk of progression and mortality over a short-to-intermediate interval.

### CMKLR1 is expressed in different categories of human fibrotic lung diseases

**Fig. 8.**
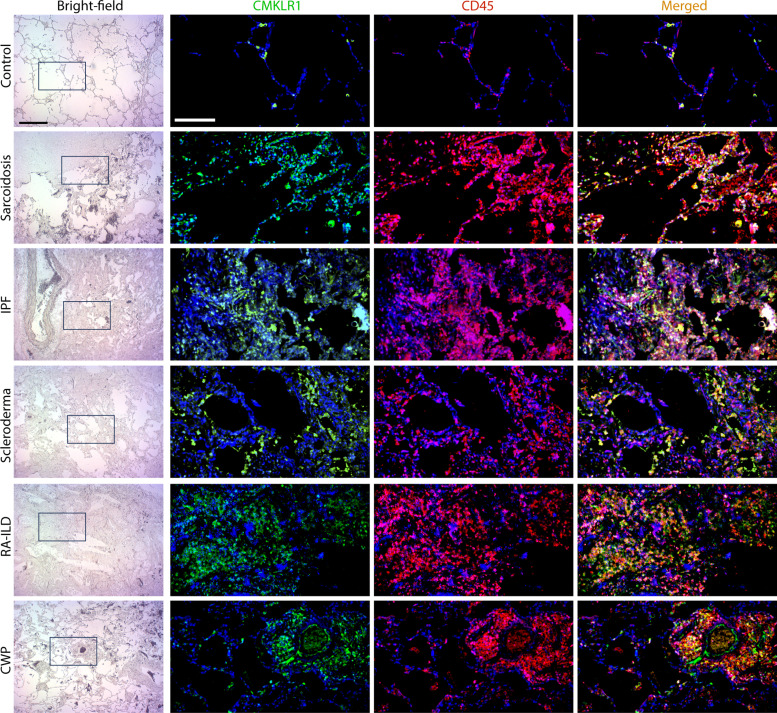
CMKLR1 expression in the lungs of patients with fibrotic lung diseases. Low-magnification bright-field and high-magnification immunostaining for CMKLR1 and CD45 demonstrate the abundance of CMKLR1-expressing leukocytes in the lungs of patients with pulmonary fibrosis associated with various etiologies. While there was a marked heterogeneity in the expression of CMKLR1 in different regions of the lungs, the example images were selected to convey CMKLR1 expression in diseased regions of the lungs. CMKLR1-expressing CD45-negative nonimmune cells were also noted across different disease categories. *n* = 3 patients for each disease category (examples from the other two patients in each category of lung diseases are shown in fig. S13). CW, coal work pneumoconiosis; RA-ILD, rheumatoid arthritis–associated interstitial lung disease. Scale bars are 500 μm in bright-field images and 200 μm in fluorescent images.

Motivated by our finding of *CMKLR1* mRNA expression by lung macrophages and BAL cells in fibrotic lung diseases, we performed an exploratory analysis to confirm histological evidence of CMKLR1 protein expression in the lungs of patients with IPF and other fibrotic lung diseases using an existing biorepository (table S1). We observed the presence of CMKLR1-expressing leukocytes in the lungs of patients with IPF, sarcoidosis, scleroderma, rheumatoid arthritis, and coal worker pneumoconiosis (*n* = 3 per disease category) (Fig. 8 and fig. S12). It is noteworthy that CMKLR1 expression was also detectable in CD45-negative cells, consistent with the scRNA-seq data, indicating the presence of *CMKLR1* expression in nonimmune cells, albeit at a lower abundance when compared to immune cells (fig. S13). Together, our exploratory histology evaluation suggests that CMKLR1 is expressed in various fibrotic lung diseases. However, it should be noted that explanted lungs usually represent features of end-stage disease, which may not be reflective of the initial pathology. Therefore, additional studies are necessary to elucidate the disease-specific patterns of CMKLR1 expression in these conditions and to ascertain its potential utility as a clinical biomarker for disease endotyping and risk stratification.

## DISCUSSION

In this study, we established *CMKLR1* as a biomarker of MDMφ enriched in patients with fibrotic lung diseases, and demonstrated the accuracy of CMKLR1-targeted PET to noninvasively monitor the accumulation of MDMφ and predict the severity of future fibrosis in the murine model of bleomycin-induced lung injury. Furthermore, we established the translational relevance of our findings by demonstrating that high expression of *CMKLR1* in BAL cells identifies an inflammatory endotype of IPF characterized by a poor prognosis and a transcriptional profile indicative of heightened inflammatory and ECM remodeling pathways.

Current therapeutic approaches to fibrotic lung diseases do not take into account the molecular endotypes and heterogeneity of the underlying pathogenic processes, which drive adverse ECM remodeling. As a result, these approaches heavily rely on long-term monitoring of functional and structural consequences, for example, by pulmonary function tests and CT, to assess disease progression and treatment response (*34*). The inability to identify distinct molecular endotypes of fibrotic lung diseases not only hampers personalized patient management but has also likely contributed to a string of clinical trials with negative outcomes as specific patient subgroups that may respond to specific treatments cannot be identified and recruited prospectively (*35*). Molecular endotyping of IPF through blood and BAL biomarkers (*36*–*39*) shows promise but is constrained by its susceptibility to systemic influences beyond the lungs in case of blood biomarkers and the invasive nature of BAL. To overcome these limitations, molecular imaging has gained increasing attention for noninvasive monitoring of key pathogenic drivers of lung inflammation-fibrosis axis (*15*, *40*).

Our comprehensive analysis of lung immune cells using large scRNA-seq datasets suggested that *CMKLR1* expression is a hallmark of nascent MDMφ, enriched in patients with fibrotic ILDs; hence, it represents a promising biomarker for noninvasive imaging of the lung inflammation-fibrosis axis. We previously described [^64^Cu]NODAGA-CG34 as a CMKLR1-targeting peptidomimetic tracer with several favorable properties, including facile radiolabeling with a high yield and molar activity, plasma stability, low plasma protein binding, and rapid clearance (*16*). Although CG34-derived peptides exhibit lower affinities to CMKLR1 (*K*_d_ ~ 200 nM) compared to chemerin (*16*), here, we demonstrated their ability to induce receptor-mediated internalization similar to the natural chemerin-derived peptides (*41*), which explains the high accumulation of [^64^Cu]NODAGA-CG34 in inflamed lungs. We demonstrated that CG34-specific binding is restricted to CMKLR1 without detectable binding to the other two receptors of chemerin, namely, CCRL2 and GPR1.

Molecular imaging strategies often encounter the limitation of targeting a single biomarker, which may not yield the same degree of specificity attained through multiparametric invasive analyses, such as immunoprofiling by flow cytometry or scRNA-seq. Recognizing this limitation, we determined the accuracy of our CMKLR1 targeting approach in detecting MDMφ by investigating the uptake of a fluorochrome-conjugated analog of [^64^Cu]NODAGA-CG34 by different immune cells at various stages of bleomycin-induced lung injury. MDMφ were the primary contributors to increased 6CF-CG34 uptake during the inflammatory phase of lung injury, accounting for ~78 and ~86% of the total uptake by the main CMKLR1-expressing cells, i.e., monocytes/macrophages and natural killer cells, at weeks 1 and 2 postbleomycin, respectively. Conversely, tissue-resident alveolar macrophages, monocytes, neutrophils, and lymphocytes showed negligible 6CF-CG34 uptake. Interstitial macrophages, on the other hand, exhibited a transient increase in 6CF-CG34 uptake during the pneumonitis phase of lung injury, peaking at week 1 (accounting for ~11% of the uptake) and declining by week 2 (~3% of the uptake). This observation may suggest CMKLR1 expression during the intermediary differentiation stage of MDMφ, as interstitial macrophages precede the differentiation of monocytes into alveolar macrophages (*42*), consistent with our scRNA-seq analysis revealing a transient *CMKLR1* expression in human lungs in their trajectory toward SPP1-Mφ. However, alternative explanations, such as the induction of CMKLR1 by resident interstitial macrophages, warrant further investigation through fate mapping studies. Consistent with our previous report (*16*), CMKLR1-expressing natural killer cells contributed significantly to the minimal baseline 6CF-CG34 uptake in noninflamed lungs, although their contribution to total uptake during lung injury was overshadowed by that of MDMφ.

[^64^Cu]NODAGA-CG34 PET at various time points after bleomycin administration closely mirrored the temporal pattern of the accumulation of CMKLR1-expressing MDMφ observed by flow cytometry. Specifically, we noted an increased [^64^Cu]NODAGA-CG34 uptake at 1 and 2 weeks after bleomycin treatment, which returned to near-baseline levels by week 4. Despite challenges inherent to PET imaging of lungs, such as respiratory motion (*43*), we achieved accurate noninvasive quantification of lung uptake of [^64^Cu]NODAGA-CG34 as evidenced by strong correlation between PET-derived and γ-counting–derived measurements. We validated the prognostic value of CMKRL1-targeted PET by establishing that [^64^Cu]NODAGA-CG34 uptake during the early stage of bleomycin-induced injury predicts the extent and spatial distribution of future pulmonary fibrosis. Together, our findings emphasize the feasibility of CMKLR1-targeted PET as a quantitative approach to detect the spatiotemporal pattern of MDMφ accumulation throughout different stages of fibrotic lung injury for noninvasive monitoring of the lung inflammation-fibrosis axis.

To confirm the translational relevance of CMKLR1 as a biomarker in fibrotic lung diseases, we demonstrated that high *CMKLR1* expression by BAL cells is associated with a distinct IPF endotype with a significantly high mortality, in particular in the short-to-intermediate timeframe, and enrichment of inflammatory and ECM remodeling pathways. This information may have major clinical implications, such as allowing for prioritizing lung transplantation or implementing more aggressive therapies in patients considered at risk of rapid deterioration. Moreover, identifying patients with inflammatory endotypes of fibrotic lung diseases may improve the design and outcome of clinical trials by allowing for recruitment of patients who are more likely to benefit from anti-inflammatory treatments and monitoring their therapeutic response. Last, our exploratory histological analysis of lung specimens suggests that CMKLR1 is a relevant inflammatory biomarker in a wide range of fibrotic lung diseases in addition to IPF, such as sarcoidosis, scleroderma, rheumatoid arthritis, and coal worker pneumoconiosis. However, to establish disease-specific patterns of CMKLR1 expression in these conditions, further investigations involving larger sample sizes are warranted.

This study has several limitations. There are relatively few preclinical models of pulmonary fibrosis and none entirely replicates the pathological complexities of the diverse diseases, which lead to lung fibrosis in humans (*44*). The widely used bleomycin-induced lung fibrosis model has been extensively validated and recapitulates several key pathological features observed in clinical disease, including the release of similar cytokines and growth factors as well as activation of relevant signaling pathways (*6*, *30*, *45*). However, the single bleomycin injection protocol does not replicate the progressive nature and spatiotemporal heterogeneity characteristic of usual interstitial pneumonia, the pathological and imaging hallmarks of IPF. In contrast, repetitive intratracheal administration of bleomycin, as a more recently developed alternative animal model, more closely recapitulates the temporal heterogeneity and long-term progression of pulmonary fibrosis (*46*, *47*). The validation of [^64^Cu]NODAGA-CG34 PET in the repetitive bleomycin instillation model may provide further biological insights and aid in distinguishing the inflammatory and ECM remodeling components of fibrotic lung injuries. CMKLR1-targeted PET may also serve as a tool for mechanistic studies, shedding light on the role of accumulated MDMφ in sustaining inflammation beyond the resolution of acute injury and driving progressive lung fibrosis. Another limitation of our study is that our analysis of the prognostic role of *CMKLR1* expression was restricted to patients with IPF and used a bulk transcriptomics approach as scRNA-seq analysis was not performed in the original study (*32*). While pulmonary fibrosis arises from various underlying etiologies, we focused on IPF as the prototypical and most common fibrotic ILD. In addition, we are not aware of other publicly available BAL transcriptomics dataset with longitudinal follow up in other fibrotic lung diseases, which limits our ability to explore the prognostic potential of CMKLR1 in other conditions. Another limitation of this study is that BAL sampling may underestimate the extent of total CMKLR1 expression, which can be detected by PET, as parenchymal CMKLR1^+^ macrophages (*16*) are not efficiently recovered by this technique. To address this limitation, we performed an exploratory study to determine CMKLR1 expression in lung tissues from patients with various causes of pulmonary fibrosis. Our findings demonstrated that CMKLR1 is widely expressed in the lungs across these etiologies. Furthermore, the current study was focused to investigate the promise of CMKLR1 as a biomarker of lung inflammation-fibrosis axis, and potential mechanistic contributions of CMKLR1 in driving adverse ECM remodeling and fibrosis require further studies. Last, because of the lack of Food and Drug Administration approval for this tracer, as an Investigational New Drug, we were unable to perform PET studies in patients. Nonetheless, the promising results obtained from [^64^Cu]NODAGA-CG34 in preclinical models of lung diseases and the high affinity of CG34 to human CMKLR1 along with the clinical relevance of CMKLR1 as a biomarker of MDMφ in patients with COVID-19 (*16*) and IPF will lay a solid foundation for future Investigational New Drug application to enable the first-in-human use of this tracer in fibrotic lung diseases.

In summary, our study highlights the potential of CMKLR1-targeted PET as a valuable tool for noninvasive monitoring of the lung inflammation-fibrosis axis through the detection of MDMφ accumulation. By providing valuable insights into the molecular processes underlying the pathogenesis of the lung inflammation-fibrosis axis, this approach holds promise for endotyping of fibrotic lung diseases and may offer opportunities for improved prognostication and optimization of individualized management strategies in a wide range of inflammatory and fibrotic lung diseases. It is noteworthy that MDMφ recruitment occurs in various inflammatory lung diseases, such as ARDS (*16*), pneumonia/pneumonitis (*48*), COPD (*49*), and cystic fibrosis (*50*), and their pathological effects depend on the context of the underlying disease rather than necessarily driving fibrosis. Consequently, the application of CMKLR1-targeted PET may extend beyond pulmonary fibrosis to various inflammatory lung diseases; hence, it is imperative to consider the unique context of the underlying disease for a better understanding of the implications of the uptake of CMKLR1-targeting tracers.

## MATERIALS AND METHODS

### Chemicals and reagents

The Supplementary Materials provide the list of major chemicals and reagents (table S2), plasmids (table S2), flow cytometry reagents (table S4), and histology antibodies (table S5).

### scRNA-seq data processing of lung immune cells

Preprocessed data from Habermann *et al.* (*21*) was downloaded from GEO accession GSE135893. Raw sequencing data from Adams *et al.* (*20*) was downloaded from GSE136831 and reprocessed; read2 5′ template-switch oligo contaminants and 3′ poly(A) contaminant sequences were trimmed using cutadapt (version 3.1), and trimmed reads were mapped to human genome GRCh38 with STARsolo (version 2.7.8.a) using Gencode release 38.

### Cell type labeling

Cells from each dataset that were identified as immune in their original analyses were isolated for downstream analyses in R (version 4.0.3) with the package Seurat (version 4.3.0.1). Cells were split into two groups based on the dataset and integrated using Seurat’s reciprocal principal components analysis (RPCA) implementation. For each cell, a “stress score” was estimated using the proportion of transcripts, which were Fos/Jun and HSP/DNAJ-family heat-shock proteins. Before the integration steps, aforementioned stress genes were blacklisted from feature selection and this stress score was regressed out during the scaling step performed before each dataset’s PCA; all downstream RPCA integration parameters were performed under default settings.

Cell type identities were resolved through an iterative and recursive process of integration, dimension reduction, graph embedding, high-resolution clustering, and expert curation. This process was repeated until all cells could be assigned to categorical groups (cell types) with positive and negative distinguishing features that could be observed consistently across multiple subjects in both datasets. For the final UMAP implementation used, principal components 1 to 16 and 18 to 23 were used; PCs 17 and 24 were avoided because their loadings were heavily influenced by metallothionines. The Seurat RunUMAP implementation was run with 15 neighbors, a minimum distance of 0.6, and repulsion strength and spread parameters both equal to 1, for 500 iterations with seed of 7. The proportion of myeloid cells in different clusters between IPF and control lungs was compared using the Mann-Whitney *U* test.

### Pseudotime analysis

The group of myeloid cells observed belonging to contiguous UMAP feature-space (monocytes, macrophage, and nonplasmacytoid dendritic cells) were subsetted for downstream analysis in Python (version 3.9.16) using the package Scanpy (version 1.9.3). Cell neighbors were recalculated with Scanpy’s scanpy.pp.neighbors implementation using the prior PCA embeddings but only the first 16 principal components (as Scanpy does not support noncontiguous sequences of PCs) with 25 neighbors per cell with the gaussian distance metric and random state of 7. The relatively large neighborhood size was chosen to reduce the impact of spurious cell-cell adjacencies. Using recalculated neighborhoods, all abundant cell types (monocytes, macrophage, and cDC2 cells) were reclustered with Scanpy’s louvain implementation with a resolution of 3 and a random seed of 7. Granular subclusters were subjected to Scanpy’s PAGA (version 1.2) implementation with a confidence threshold of 0.65. Intersubpopulation edges pruned via PAGA were also pruned in the corresponding cell-cell graph to prevent spurious connections in feature space before pseudotime estimations. Pseudotime estimates for each trajectory were performed via the Scanpy external wrapper for Palantir (version 1.0.1) using the pruned cell-level adjacency matrix, 15 diffusions components, and 1000 waypoints per trajectory.

Pseudotime estimation results were then subjected to downstream analysis in R (this time, version 4.3.0) using the package tradeSeq (version 1.13.01) to fit GAMs of variable genes to each pseudotime ordering trajectory. To downsample cells without exacerbating biases in representation along pseudotime, cells from each trajectory were broken into 1001 bins in the pseudotime distance range between 0 and 1 by 0.001 increments, with a maximum of three cells randomly sampled per bin (seed = 7). Among these cells, the top 1000 variable genes were identified using Seurat’s (version 4.3.0.1) FindVariableFeatures implementation with otherwise default parameters. For tradeSeq’s fitGam implementation, both trajectories had GAMs fit in parallel, where cells belonging to both trajectories were assigned a 0.5/0.5 probability for belonging to either trajectory, with seven knots per model using the negative binomial fit, with dataset treated as a random covariate. While only downsampled cells and highly variable genes were included in the GAM analysis, an offset value to normalize each cell was calculated using the log transformed total number of transcripts across all genes.

### Secondary analysis of scRNA-seq from IPF and control upper versus lower lobes

Processed scRNA-seq count data of control and IPF lung tissue described in Morse *et al.* (*25*) was downloaded from GEO accession GSE128033. Cell barcodes with at least 700 transcripts and less than 15% of transcripts of mitochondrial origin were used for analysis. Immune cells were isolated and cell type labels and approximate UMAP coordinates were projected onto them using the Seurat implementation MapQuery(), with the integrated immune dataset as reference. Marker genes were compared between reference and query datasets to confirm label-transfer reliability.

### Cell culture

CHO-K1 cells were grown in complete growth media consisting of Ham’s F-12K (Kaighn’s) medium supplemented with 10% fetal bovine serum and 1% penicillin/streptomycin. Resident peritoneal cells were obtained from C57BL/6J mice, as previously described (*16*), cultured in RPMI medium supplemented with 10% fetal bovine serum and 1% penicillin/streptomycin.

### Transient transfection

CHO-K1 cells at 70 to 90% confluency were subjected to transient transfection with mouse CMKLR1, mouse GPR1, or mouse CCRL2 plasmids using Lipofectamine 3000 following the manufacturer’s instructions.

### Stable transfection

CHO-K1 cells at 70 to 90% confluency were transfected with human CMKLR1 and G_α15_ plasmids using Lipofectamine 3000 following the manufacturer’s instructions. Transfected cells were next treated with selection antibiotics [geneticin (400 μg/ml) and hygromycin B (800 μg/ml)] and grown for ~2 weeks. Then, individual cell colonies were subcultured and grown until sufficient numbers were available to evaluate the expression of the transfected gene by flow cytometry.

### Radiolabeling

NODAGA-CG34 was radiolabeled and characterized as previously reported (*16*). Radio–high-performance liquid chromatography was performed to ensure a radiochemical purity of >95% for [^64^Cu]NODAGA-CG34 before its use in all in vitro and in vivo experiments.

### Mouse model of fibrotic lung injury

Animal experiments were conducted in accordance with a protocol approved by the University of Pittsburgh Institutional Animal Care and Use Committee. To induce fibrotic lung injury, bleomycin (1.5 U/kg) in 60 μl of phosphate-buffered saline (PBS) was intratracheally administered to C57BL/6J mice (~9 to 12 weeks old, Jackson Laboratory, strain no. 000664). Control mice were intratracheally injected with 60 μl of PBS. Mice were euthanized at 1, 2, or 4 weeks after the induction of lung injury or if their weight loss exceeded 25% of the initial body weight.

### [^64^Cu]NODAGA-CG34 PET/CT

At specified time points following administration of bleomycin or PBS, mice were injected intravenously with [^64^Cu]NODAGA-CG34 (6.76 ± 0.07 MBq). To address tracer specificity, a co-injection of [^64^Cu]NODAGA-CG34 with ~100-fold molar excess of nonradiolabeled NODAGA-CG34 was performed in a cohort of mice at 2 weeks after bleomycin administration. At 90 min postinjection, static PET (~10 min) and low-resolution CT (projections, 180; exposure, 140 ms; rotation, 180°; voltage, 80 kV; current, 500 μA; and field of view, 78.5 × 100 mm) were performed using a Siemens Inveon scanner. Kinetic PET/CT from ~5 to 90 min postinjection was performed in a cohort of mice using the same parameters. High-resolution CT in the longitudinal study was performed using the following parameters: projections, 220; exposure, 800 ms; rotation, 220°; voltage, 60 kV; current, 500 μA; and field of view, 30.2 × 31.1 mm.

Regions of interest were drawn over the left and right lungs of attenuation-corrected PET images, and the uptake of [^64^Cu]NODAGA-CG34 in the left and right lungs was averaged and quantified as the mean and maximal percentage of injected dose per ml of tissue (%ID/ml_mean_ and %ID/ml_max_) using IRW software. To quantify the blood pool concentration of [^64^Cu]NODAGA-CG34 by PET, a spherical region of interest was drawn inside of the left ventricle. Biodistribution was performed by γ-counting (Wizard^2^, PerkinElmer) of harvested organs, and data were presented as decay-corrected percentage of injected dose per gram tissue (%ID/g). Multiplanar reconstructions were performed using software to align PET/CT with autoradiography and histology images.

### [^64^Cu]NODAGA-CG34 autoradiography

Mice were euthanized following PET/CT, and their lungs were inflated with 1 ml of optimal cutting temperature compound (OCT). Subsequently, the lungs were embedded in OCT and frozen for cryosectioning (10 μm thickness) within ~24 hours of tracer injection. After an overnight exposure of tissues to phosphor screens (GE, BAS-IP SR2025 Super VivoQuant Resolution), screens were scanned at a resolution of 100 μm using a Sapphire Biomolecular Imager, as previously described (*16*).

### Mouse lung immunostaining

After euthanasia, the pulmonary circulation of mice was perfused with ~5 ml of PBS through a right ventricular puncture. The lungs were then inflated by 1 ml of intratracheal OCT and stored frozen. Cryosections of 10 μm thickness were prepared. Tissues were fixed with a cold methanol-acetone mixture (1:1). Next, primary antibodies at a dilution of 1:200 were applied to the sections and incubated overnight at 4°C. Following PBS washes, fluorescently conjugated secondary antibodies were added and incubated for 2 hours at 4°C. Slides were mounted with ProLong Gold Antifade Mountant containing 4′,6-diamidino-2-phenylindole (DAPI) and visualized using an Axio Vert microscope (Zeiss).

### Binding/uptake of 6CF-CG34 and 6TAM-CG34 by peritoneal macrophages

Peritoneal cells were incubated in indicator-free RPMI in the presence of increasing concentrations of 6CF-CG34 or 6TAM-CG34, both with and without co-incubation with unlabeled Chem_145–157_ (high-affinity CMKLR1 ligand derived from the amino acids 145 to 157 of chemerin; peptide sequence, P-H-S-F-Y-F-P-G-Q-F-A-F-S-COOH; 2.5 μM) (*16*), for 1 hour at 37°C. After a single wash with PBS, flow cytometry was performed using LSR II Flow Cytometer (BD Biosciences) and analyzed by FlowJo software version 10.7.2 (BD Biosciences). Total, nonspecific, and specific saturation binding curves were generated using GraphPad Prism software.

### Competitive binding/uptake assay of 6CF-CG34 or 6TAM-CG34 versus NODAGA-CG34 in peritoneal macrophages

Peritoneal cells were incubated in indicator-free RPMI in the presence of 6CF-CG34 or 6TAM-CG34 (50 nM) with increasing concentrations of NODAGA-CG34 (10^−10^ M to 10^−5^ M) for 1 hour at 37°C. After a single wash with PBS, flow cytometry was performed using LSR II Flow Cytometer and analyzed by FlowJo software. Competitive binding curves were generated using GraphPad Prism Software.

### Binding/uptake of fluorescently tagged CG34 peptides by CHO-K1 cells transiently expressing mouse or human CMKLR1, mouse GPR1, or mouse CCRL2

CHO-K1 cells transiently expressing mouse CMKLR1 (mCMKLR1), human CMKLR1 (hCMKLR1), mouse GPR1 (mGPR1), or mouse CCRL2 (mCCRL2) were incubated in indicator-free RPMI in the presence of increasing concentrations of 6CF-CG34 or 6TAM-CG34 with or without co-incubation with unlabeled CG34 (2.5 μM) for 1 hour at 37°C. After a single wash with PBS, flow cytometry was performed using a FACSCalibur or LSR II Flow Cytometer (BD Biosciences) and analyzed by FlowJo software. Total, nonspecific, and specific saturation binding curves were generated using GraphPad Prism Software.

### Internalization of 6TAM-CG34 by mouse CMKLR1

CHO-K1 cells transiently expressing mCMKLR1 or nontransfected cells grown on glass coverslips coated with fibronectin were incubated in indicator-free RPMI with 6TAM-CG34 (250 nM) with or without co-incubation with CG34 (10 μM) for 1 hour at 37°C. After a single wash with PBS, cells were incubated with Alexa Fluor 647–conjugated wheat germ agglutinin (2 μg/ml) for 20 min at 4°C to label the cell membrane. Subsequently, cells were fixed with formalin and coverslips were mounted onto glass slides with ProLong Gold Antifade Mountant containing DAPI and photographed with an Axio Vert microscope.

### Flow cytometric immunophenotyping of murine lung cells

Following euthanasia of bleomycin- or PBS-treated mice, the lungs were perfused with PBS. Mechanical dissociation of the lungs was performed using a gentleMACS Dissociator (Miltenyi) as described previously (*16*). The cells were then divided into separate tubes and incubated in indicator-free RPMI in the absence or presence of 50 nM 6CF-CG34, with or without co-incubation with 10 μM Chem_145–157_, for 1 hour at 37°C. After a PBS wash, nonspecific binding was blocked using 1% bovine serum albumin and Fc block (2 μl per sample) before incubation with a mixture of antibodies (0.5 μl per sample) and DAPI (5 μl of 1 μg/ml per sample) for 0.5 hour at 4°C. Last, cells were washed and fixed with 4% formalin for 0.5 hour at room temperature. Flow cytometry was performed using an LSR II Flow Cytometer and analyzed by FlowJo software. We defined distinct lung macrophages based on their differential expression of three surface markers, i.e., alveolar macrophages (CD11b^lo^/CD11c^hi^/SiglecF^hi^), interstitial macrophages (CD11b^hi^/CD11c^lo^/SiglecF^lo^), and MDMφ (CD11b^hi^/CD11c^hi^/SiglecF^int^), as previously confirmed by lineage tracing studies (*6*, *7*). The number of 6CF-CG34 molecules bound to/internalized by cells was calculated based on a standard curve generated using the FITC Easy Calibration Kit (Spherotech) according to the manufacturer’s instructions. Because of the low recovery by mechanical dissociation, dendritic cells were excluded from the subsequent analysis (*16*).

### Hydroxyproline assay

The lungs of euthanized mice were frozen on dry ice and stored at −80°C. Hydroxyproline contents of the left lungs were measured using a commercially available kit following the manufacturer’s instruction (Sigma-Aldrich).

### Transcriptomics analysis of BAL cells in patients with IPF

A secondary analysis of a previously published (*32*) and publicly available BAL transcriptome dataset (GSE70867) in patients with IPF (*n* = 176) and control participants (*n* = 20) was conducted. The characteristics of the patients and controls as well as the methodology for microarray bulk mRNA transcriptomics were described in the original paper (*32*). Patients were recruited from three sites: Freiburg, Germany (*n* = 62); Leuven, Belgium (*n* = 64); and Siena, Italy (*n* = 50). BAL samples were collected before the initiation of antifibrotic or anti-inflammatory medications. To account for variations across study sites, batch effect adjustment was performed using the ComBat function [in SVA R package version 3.46.0 (*51*, *52*), RStudio statistical system, version R 4.2.2]. Differential gene expression analysis was conducted (using limma package version 3.54.2) (*53*) between patients with below-median and higher-than-median *CMKLR1* expression in BAL cells. Next, we conducted a pathway enrichment analysis using the enrichKEGG function from the clusterProfiler R package (version 4.7.1.003). All genes were mapped to Entrez IDs. Our pathway analysis was limited to genes that were significantly overexpressed (adjusted *P* < 0.01 by Benjamini-Hochberg procedure) in patients with higher-than-median versus below-median *CMKLR1* expression.

To determine the prognostic role of *CMKLR1* expression in predicting survival of patients with IPF, we performed Kaplan-Meier analysis after stratifying patients into tertiles of *CMKLR1* expression. Next, we studied whether *CMKLR1* expression level is an independent predictor of survival using multivariable Cox proportional hazards regression analysis (SPSS) by adjusting for potential confounders, age, sex, study sites, and GAP score. Further, to evaluate whether *CMKLR1* expression provides incremental prognostic information compared to the risk stratification achieved by the GAP index, we performed Kaplan-Meier analysis after stratifying patients into low versus high GAP index groups (0 to 4 versus 5 to 8, respectively). Time-varying ROC curves were plotted to compare the potential of *CMKLR1* expression and GAP index in predicting mortality over the course of 1 to 4 years after BAL. Kaplan-Meier analyses were also performed after stratifying patients into tertiles of *CCR2* and *SPP1* expression.

### Human lung immunostaining

Deidentified paraffin-embedded lung tissues from patients with different types of fibrotic lung diseases and controls (table S1) were obtained from the University of Pittsburgh Biorepository Core. Signed informed consents were received before organ procurement for participation in the biorepository and secondary research use under a protocol approved by the University of Pittsburgh Institutional Review Board. A determination of Not Human Subject research was made for this specific study (no. 23070112) by the University of Pittsburgh Institutional Review Board, which involved secondary analysis of deidentified lung tissues sections that were not collected specifically for this study. Immunostaining was performed after antigen retrieval achieved by heating 5-μm-thick tissues in sodium citrate buffer (10 mM) to 90°C for 20 min. The primary antibodies, anti-CMKLR1 (1:2000 dilution) and anti-CD45 (1:500 dilution), were applied and incubated at 4°C overnight. Following PBS washes, secondary antibodies (1:200 dilution) were applied to the tissues and incubated for 120 min at 4°C. Slides were mounted with ProLong Gold Antifade Mountant containing DAPI and photographed using an Axio Vert microscope.

### Statistical analysis

Statistical analysis of human lung scRNA-seq and BAL transcriptomics data are described in the relevant section. Statistical analyses of murine data were performed using Prism 9 (GraphPad). The data are shown as the mean ± SEM. A Student’s *t* test was performed to compare the mean values between two groups. One-way analysis of variance (ANOVA), followed by Fisher’s exact post hoc test, was used to compare the mean values in >2 groups. Correlations between variables were analyzed by the Pearson test. Statistical significance was considered as *P* < 0.05.
