# Supplementary material for: Noninvasive assessment of the lung inflammation-fibrosis axis by targeted imaging of CMKLR1
Source: Sci Adv. 2024 Jun 19;10(25):eadm9817. doi: 10.1126/sciadv.adm9817 (PMC11186491; doi:10.1126/sciadv.adm9817)
Supplement: Supplementary file 1 — Figs. S1 to S13 Tables S1 to S5 Legends for data files S1 to S5 [file sciadv.adm9817_sm.pdf]

Science Advances
AAAS

Supplementary Materials for

Noninvasive assessment of the lung inflammation-fibrosis axis by targeted imaging of CMKLR1

Philip Z. Mannes *et al.*

Corresponding author: Sina Tavakoli, sit23@pitt.edu

Sci. Adv. **10**, eadm9817 (2024)
DOI: 10.1126/sciadv.adm9817

The PDF file includes:

Figs. S1 to S13
Tables S1 to S5
Legends for data files S1 to S5

Other Supplementary Material for this manuscript includes the following:

Data files S1 to S5

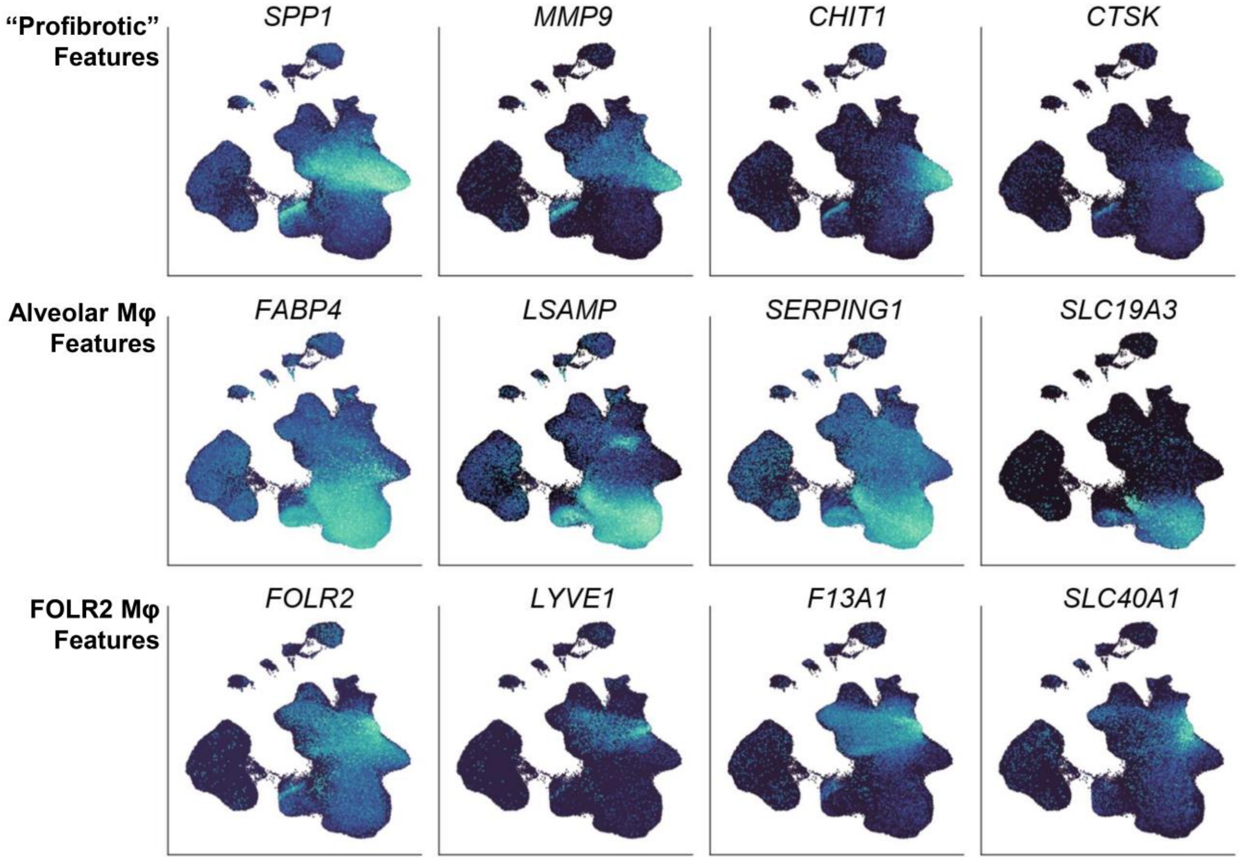

Figure S1. UMAP representation of the expression of characteristic markers of profibrotic macrophages (SPP1-Mφ), resident alveolar macrophages (Alv-Mφ), and FOLR2^{+} macrophages (FOLR2-Mφ). FABP4^{Int}-Mφ were defined as a subpopulation of macrophages with shared expression of Alv-Mφ features (e.g., *FABP4*, *LSAMP*, *SERPING1*, *SLC19A3*), albeit with a lower relative intensity, and SPP1-Mφ (e.g., *SPP1*, *MMP9*, *CHIT1*, *CTSK*).

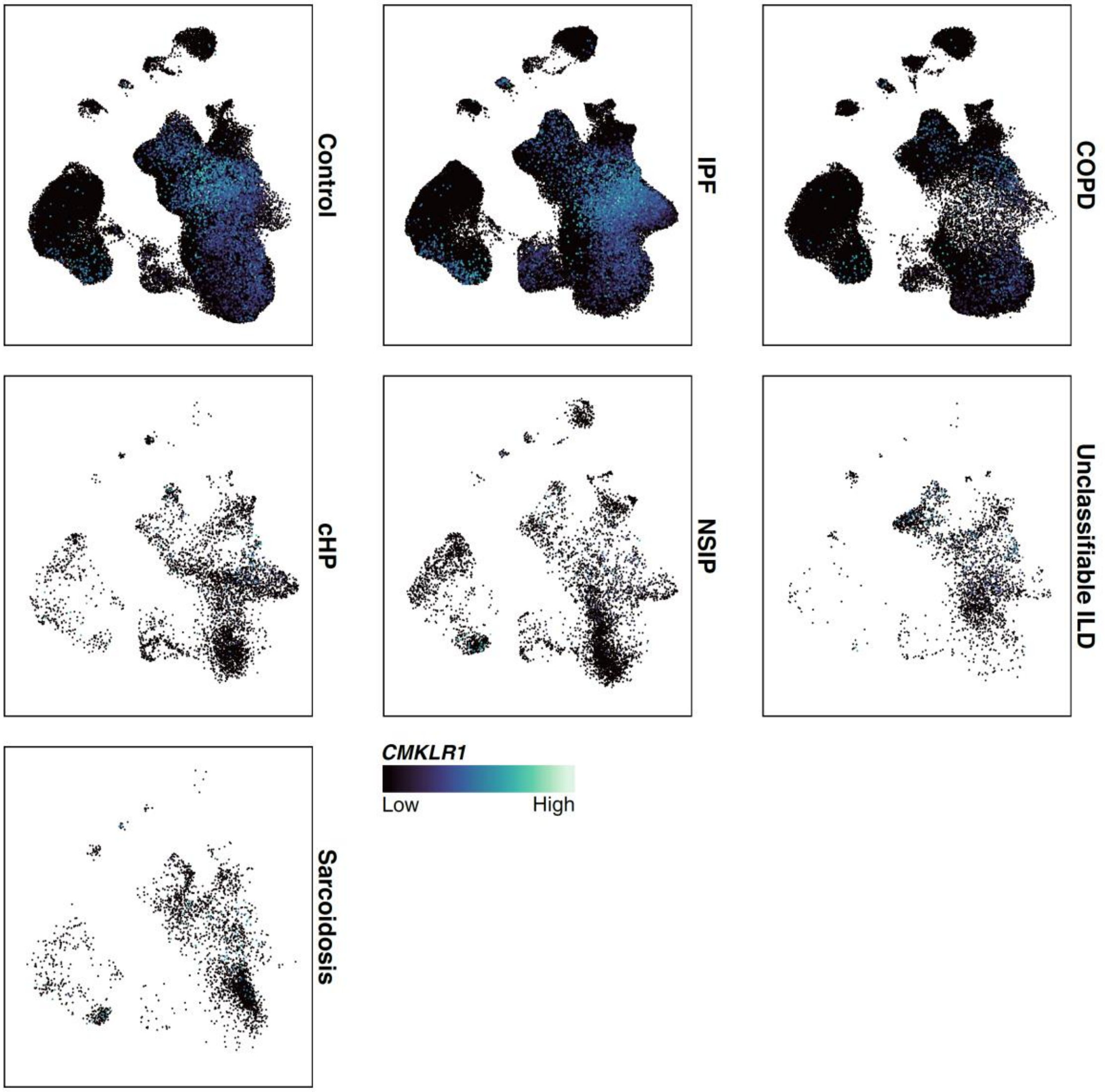

Figure S2. Exploratory analysis of *CMKLR1* expression in different ILD entities and COPD. Disease-specific UMAP plots from the scRNA-seq datasets demonstrated in Fig. 1 suggest a preferential expansion of *CMKLR1*-expressing macrophages in IPF compared to other ILD entities and COPD. N = 38 (control), 43 (idiopathic pulmonary fibrosis [IPF]), 18 (chronic obstructive pulmonary disease [COPD]), 2 (chronic hypersensitivity pneumonitis [cHP]), 3 (nonspecific interstitial pneumonia [NSIP]), 1 (unclassifiable interstitial lung disease [ILD]), and 2 (sarcoidosis).

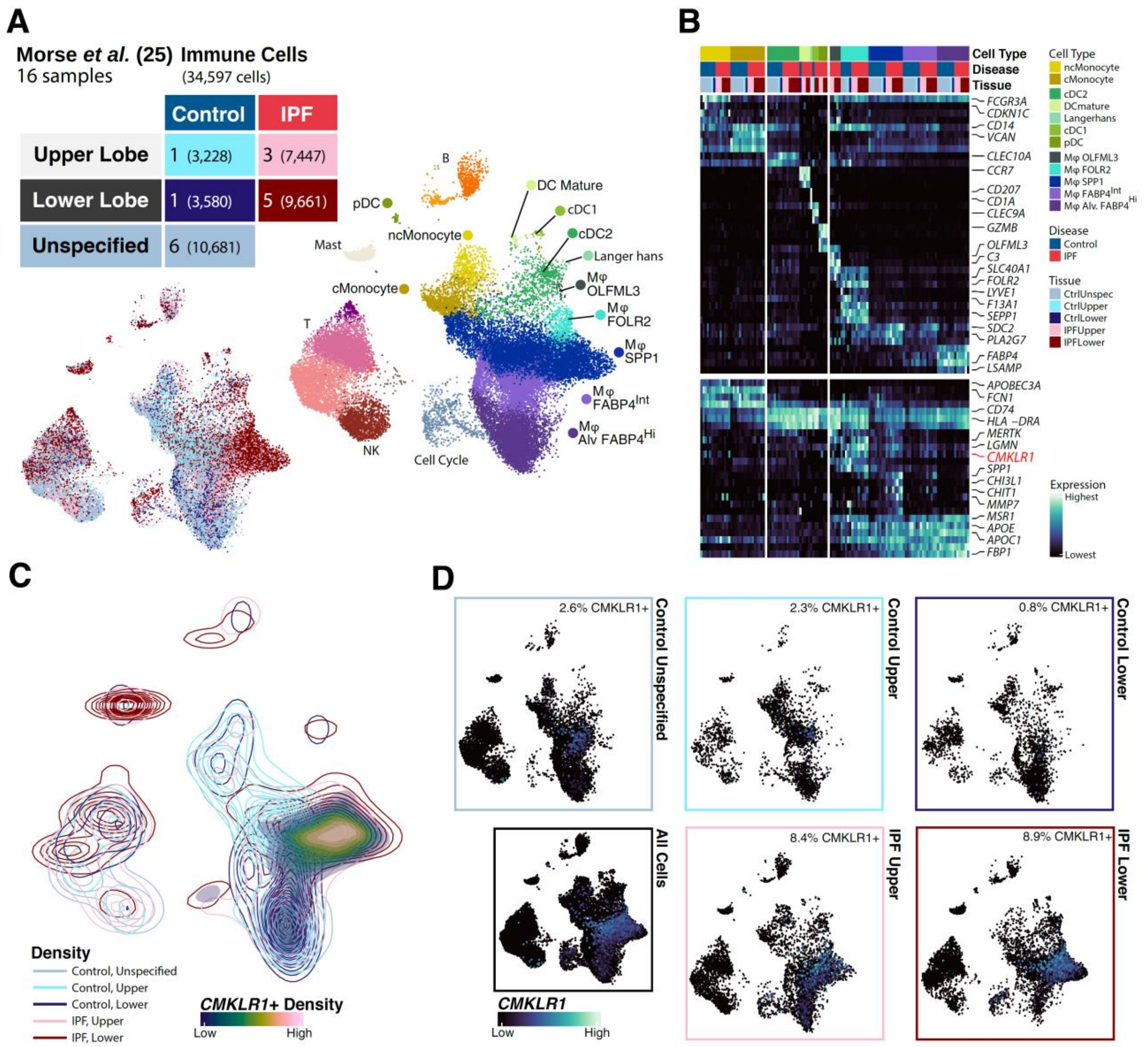

Figure S3. Secondary analysis of immune scRNA-seq analysis in control and IPF lungs. (**A**) An overview of the Morse *et al*. (25) dataset. Above: table with the number of unique samples (and cells) per condition; below left: UMAP of cells labeled by condition; below right: UMAP of cells labeled by predicted cell type. (**B**) Heatmap of marker genes across cell types. Each column represents the average gene expression per cell type for one sample, gene expression is normalized between 0 and 1. (**C**) Plot of cell densities in UMAP space: density contour lines are shown for different sample conditions, the density of *CMKLR1* expressing cells is represented by a gradient fill effect. (**D**) UMAPs of normalized *CMKLR1* expression across different conditions. For each condition, the proportion of immune cells observed with *CMKLR1* expression is shown.

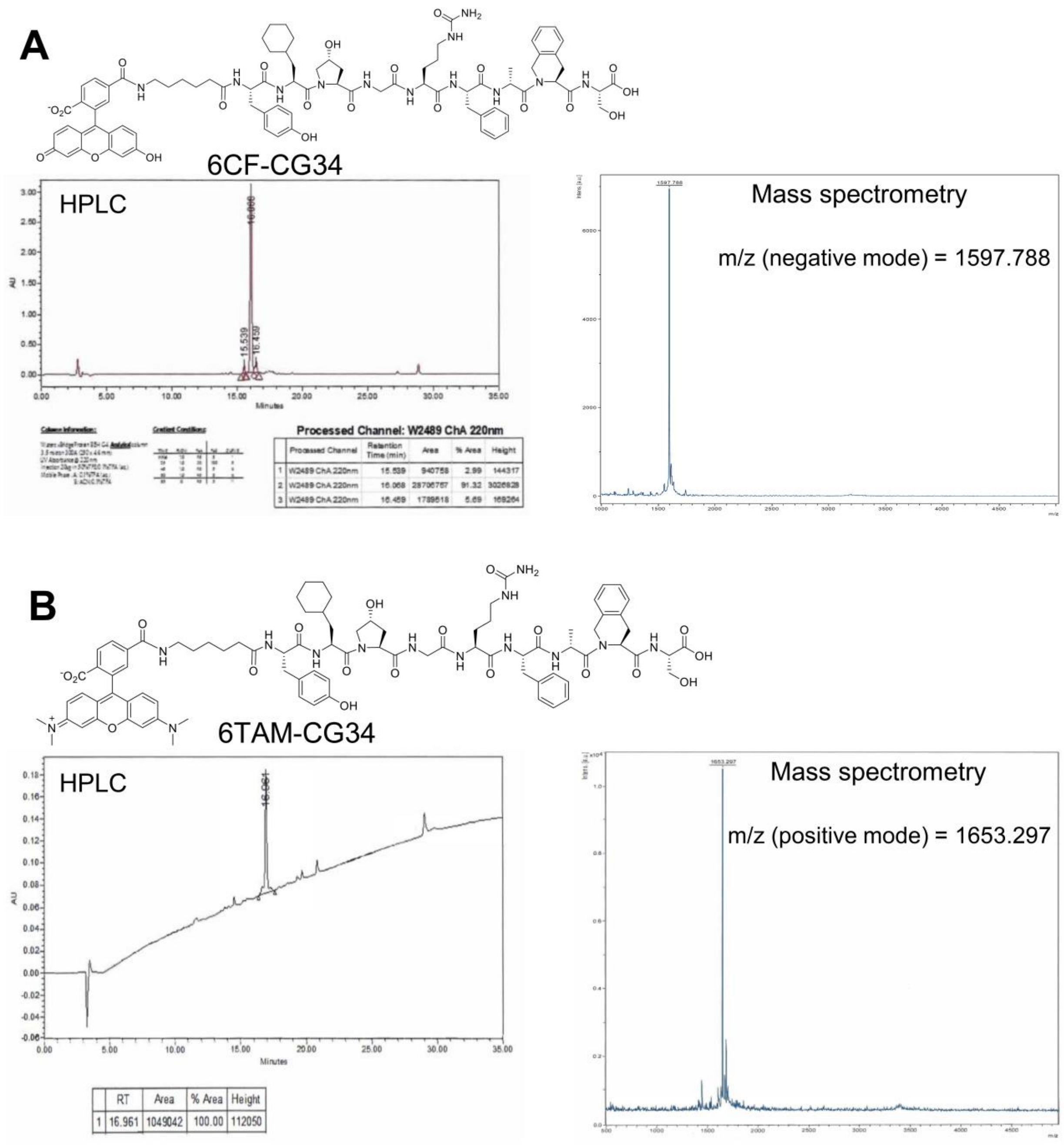

Figure S4. Characterization of 6CF-CG34 and 6TAM-CG34. The purity and molecular mass of fluorescent analogs of CG34, including 6CF-CG34 (**A**) and 6TAM-CG34 (**B**), were confirmed by high-performance liquid chromatography (HPLC) and high-resolution mass spectrometry.

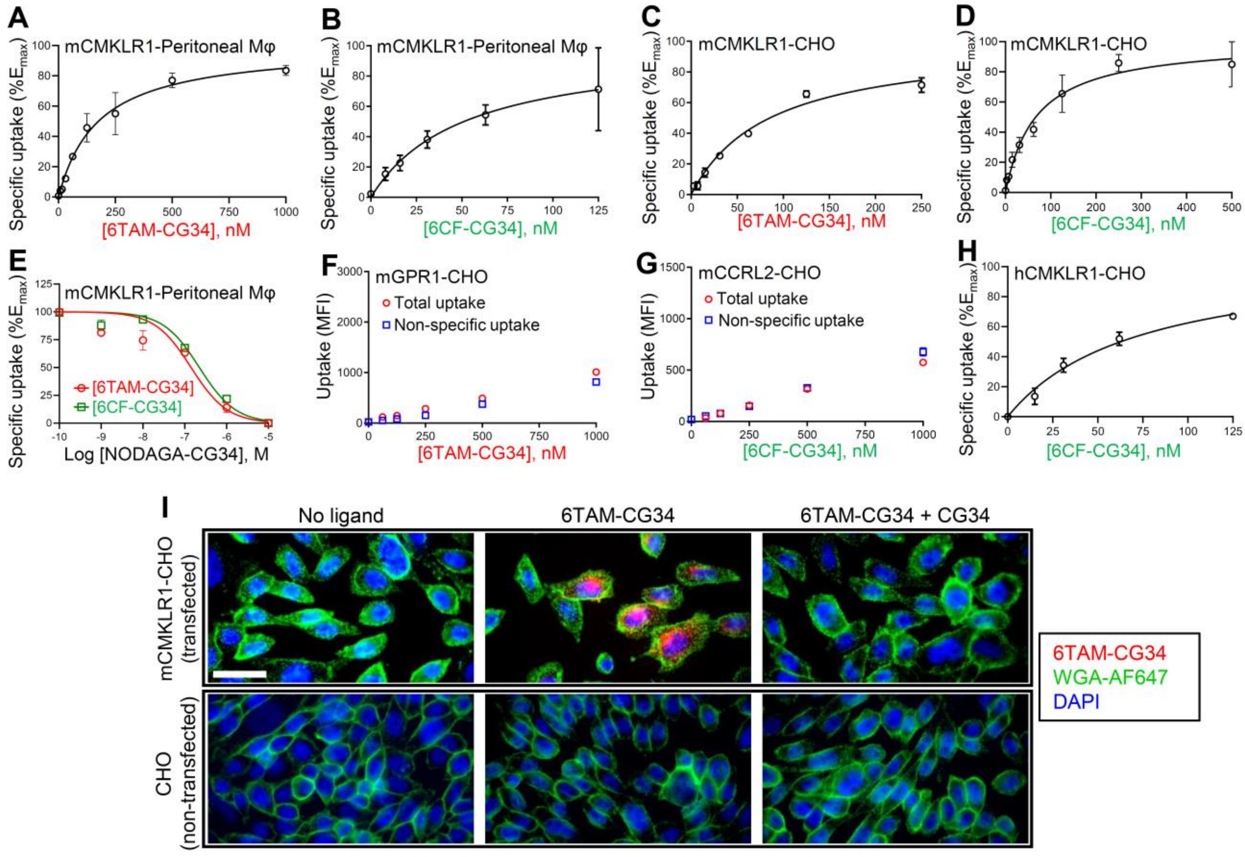

Figure S5. Potency and selectivity of 6CF-CG34 and 6TAM-CG34 for CMKLR1. 6CF-CG34 and 6TAM-CG34 showed comparable binding and uptake in both mouse CMKLR1$^+$ peritoneal macrophages (**A-B**) and CHO cells transiently expressing mCMKLR1 (**C-D**). NODAGA-CG34 displaces 6CF-CG34 and 6TAM-CG34 in a competitive uptake assay with CMKLR1$^+$ peritoneal macrophages (**E**). No significant binding/uptake was observed for fluorescent CG34 analogs to the other two chemerin receptors, i.e., GPR1 (**F**) and CCRL2 (**G**). 6CF-CG34 demonstrates potency at hCMKLR1 similar to that of mCMKLR1 (**H**). 6TAM-CG34 (250 nM) is internalized by CHO cells transiently expressing mCMKLR1 (**I**). The specificity of 6TAM-CG34 internalization to mCMKLR1 is confirmed by co-incubation with non-fluorescent CG34 (10 μM). No 6TAM-CG34 signal is observed in non-transfected CHO cells. The cell membrane was stained with wheat germ agglutinin conjugated to Alexa Fluor 647 (WGA-AF647). Scale bar: 40 μm.

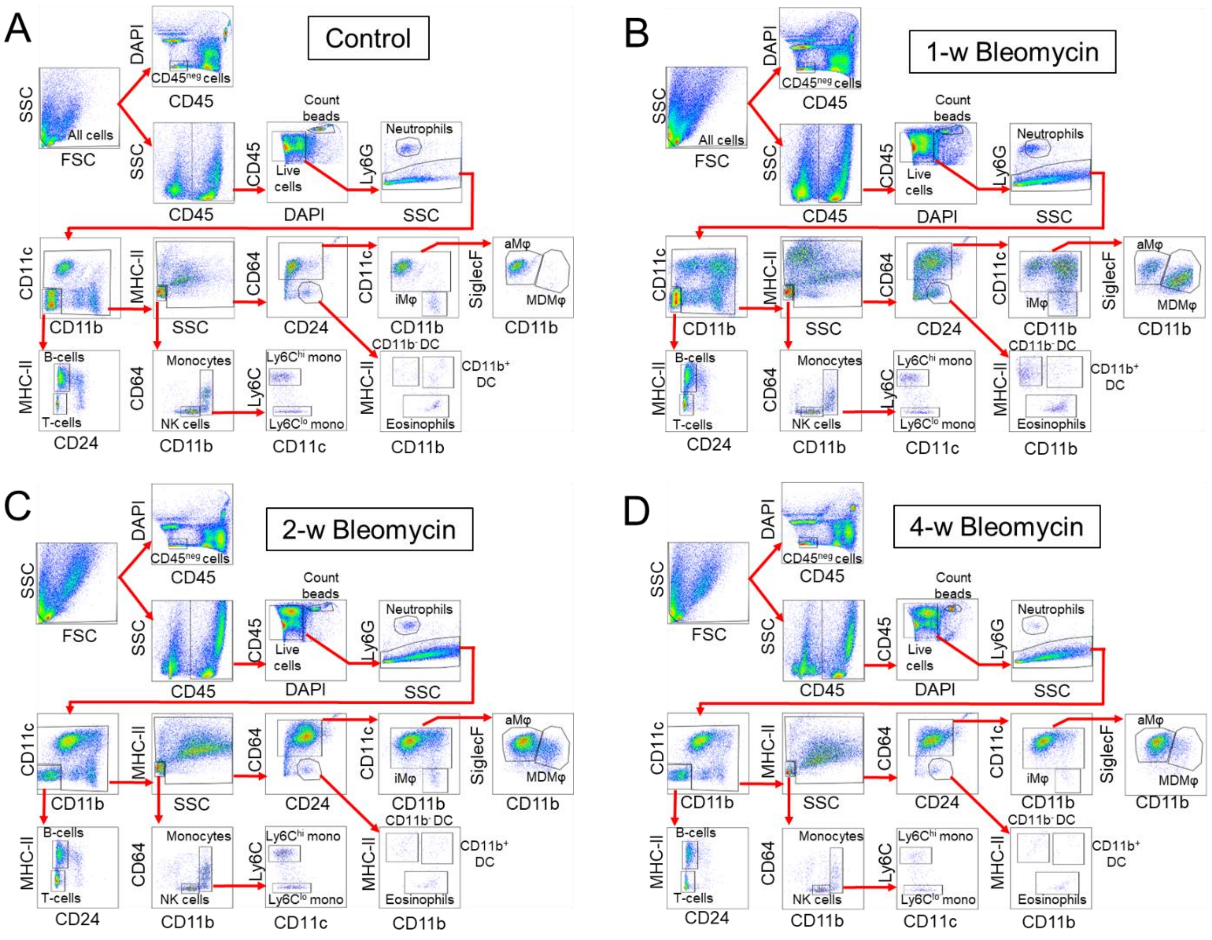

Figure S6. Gating strategy for flow cytometric analysis of lung immune cells. The gating strategy used to identify the specific leukocyte populations in control mice (**A**) and at one (**B**), two (**C**), or four (**D**) weeks following intratracheal bleomycin administration. aMφ: alveolar macrophages; CD11b$^-$ DC: CD11b$^-$ dendritic cells; CD11b$^+$ DC: CD11b$^+$ dendritic cells; iMφ: interstitial macrophages; Ly6C^{hi} mono: LyC6^{hi} monocytes; Ly6C^{lo} mono: Ly6^{lo} monocytes; MDMφ: monocyte-derived macrophages; NK cells: natural killer cells.

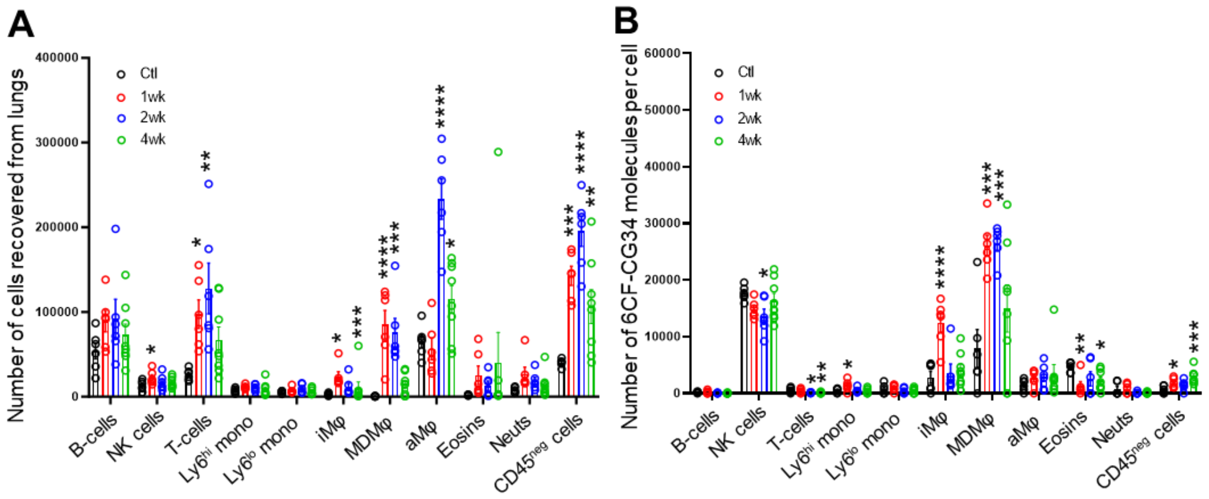

Figure S7. Flow cytometric quantification of cell count and uptake of 6CF-CG34 by lung leukocyte subsets. Following treatment with bleomycin or PBS (i.e., control), the absolute cell count recovered from the lungs (**A**) and the number of 6CF-CG34 molecules per cell (**B**) were determined with flow cytometry. aMφ: alveolar macrophages; Eosins: eosinophils; iMφ: interstitial macrophages; Ly6C^{hi} mono: LyC6^{hi} monocytes; Ly6C^{lo} mono: Ly6^{lo} monocytes; MDMφ: monocyte-derived macrophages; NK cells: natural killer cells; Neuts: neutrophils. *P*-values: * < 0.05; ** < 0.01; *** < 0.001; **** < 0.0001. Statistical significance between groups was calculated using a one-way ANOVA with a post-hoc two-tailed Fisher's exact test.

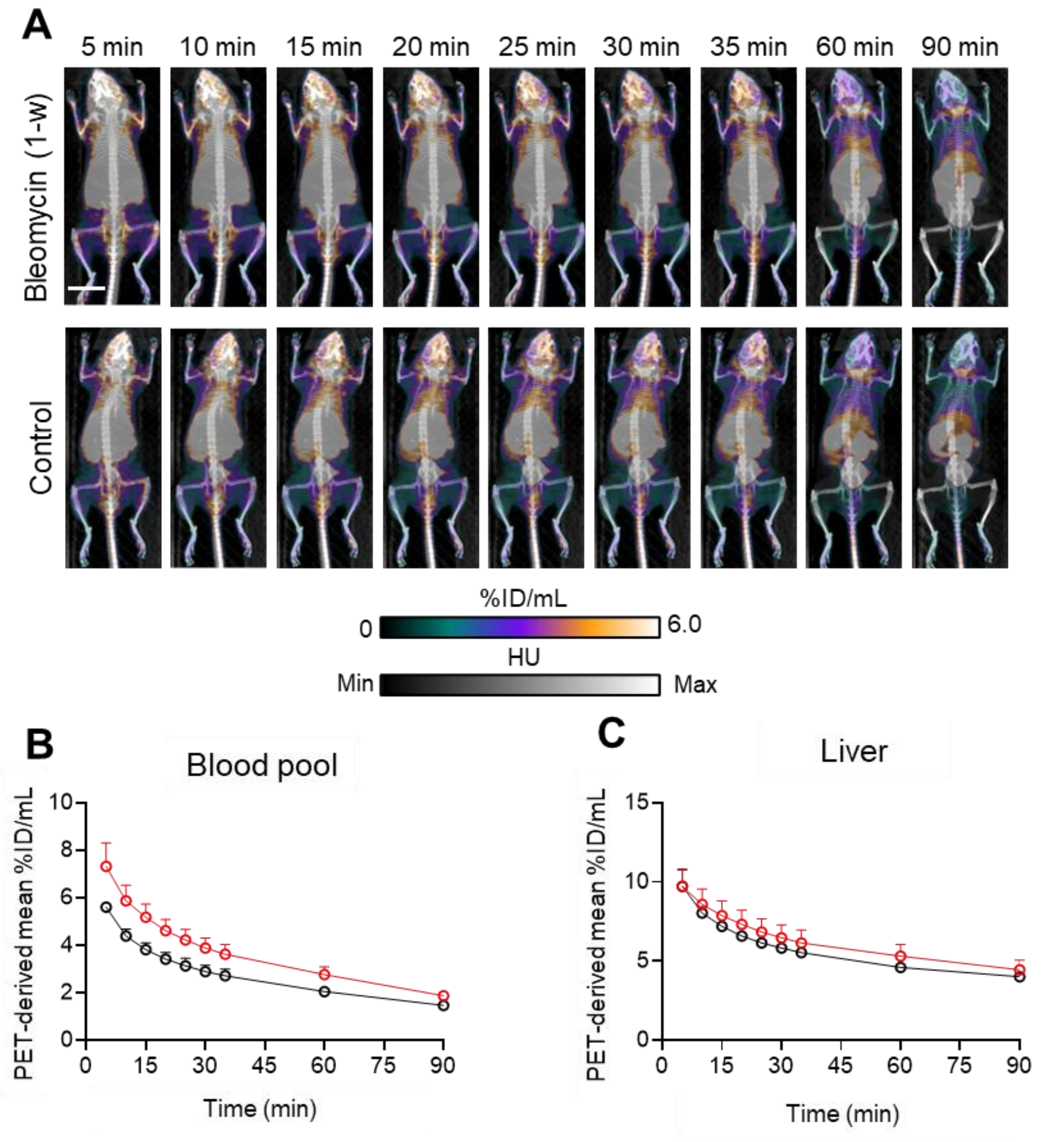

Figure S8. Kinetics of PET-derived [^{64}Cu]NODAGA-CG34. (**A**) Dynamic PET scans were performed to evaluate radiotracer accumulation in various organs as a function of time (scale bar: 15 mm). In addition to a high ratio of [^{64}Cu]NODAGA-CG34 uptake in the lungs of mice treated with bleomycin (1-week) *vs.* that of controls, there was clearance of [^{64}Cu]NODAGA-CG34 from lung adjacent organs (**B** = blood pool and **C** = liver). N = 3 for bleomycin-treated mice and 2 for control mice). Data are shown as the mean ± SEM.

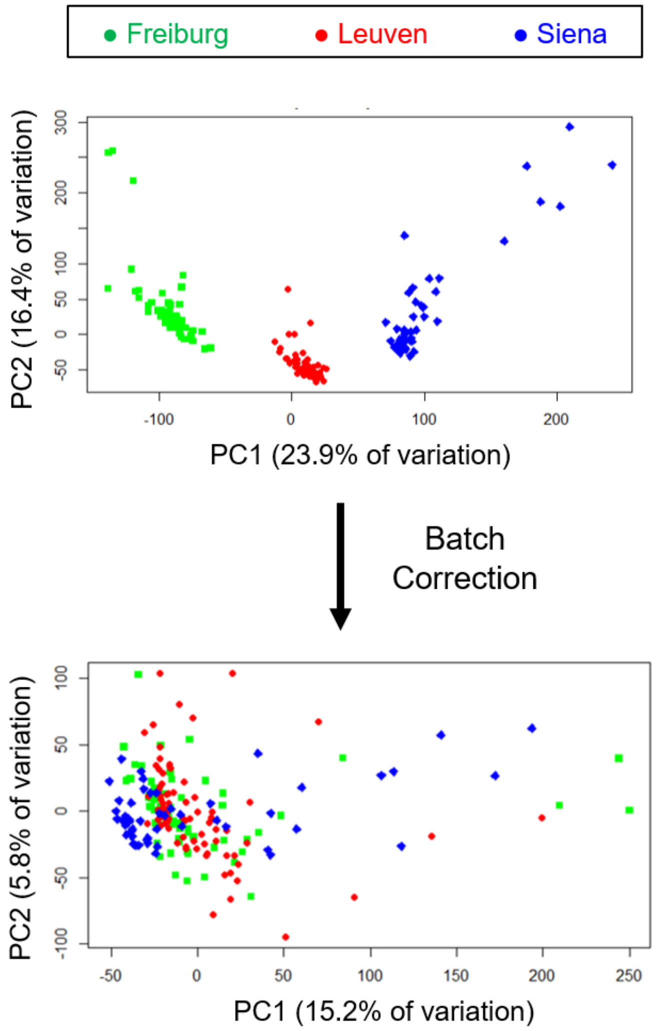

Figure S9. Adjustment for batch effect in transcriptomics analysis of BAL samples from patients with IPF. Batch effect adjustment was performed to account for variations in transcriptomics analysis of BAL specimens from patients with IPF based on the three different study sites: Freiburg, Leuven, and Siena. The top score plot shows the distribution of samples based on the first two principal components (PC1 and PC2) of the principal component analysis before applying the batch effect correction, while the bottom panel displays the score plot after applying ComBat function for batch effect correction. The application of ComBat function minimized the influence of batch effects, resulting in reduced variability based on the study sites.

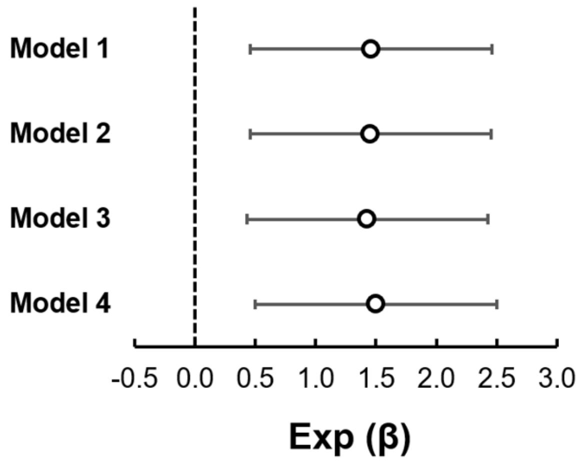

Figure S10. Cox proportional hazards regression analysis of *CMKLR1* expression as a predictor of survival in IPF. The summary of β-coefficients (displayed as e^{β} ± confidence intervals) from Cox proportional hazards regression models confirmed that BAL expression of *CMKLR1* is a statistically significant predictor of mortality after adjusting for potential confounders, including age, sex, study sites, and GAP index. Model 1: unadjusted; Model 2: adjusted for age and sex; Model 3: adjusted for age, sex, and study sites; and Model 4: adjusted for age, sex, study sites, and GAP index.

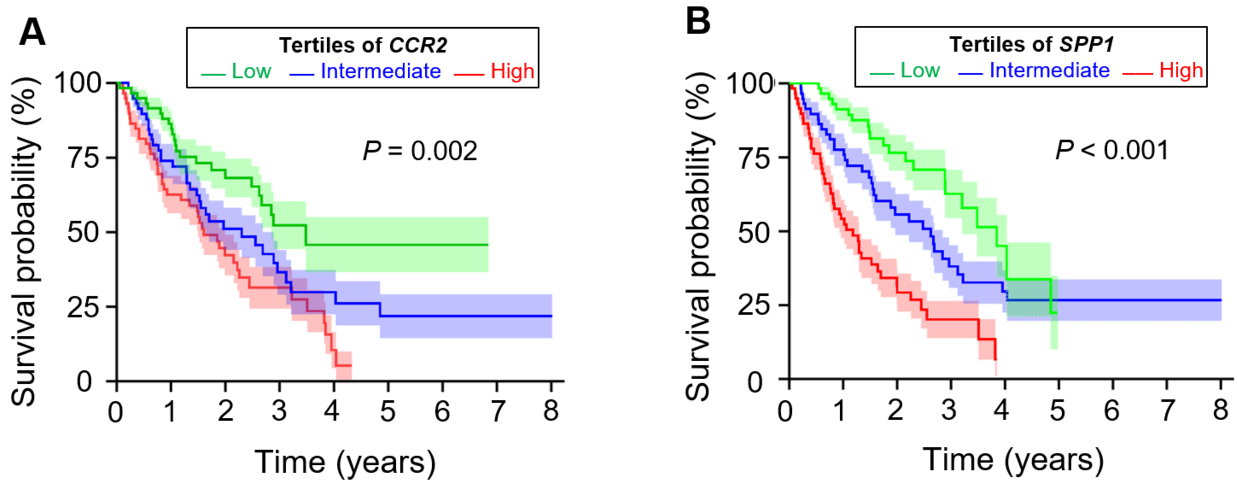

Figure S11. Survival analysis of patients with IPF based on the expression of *CCR2* and *SPP1*. Kaplan-Meier survival curves demonstrate that higher expression of *CCR2* **(A)** and *SPP1* **(B)** by BAL cells is associated with a significantly worse survival in IPF patients.

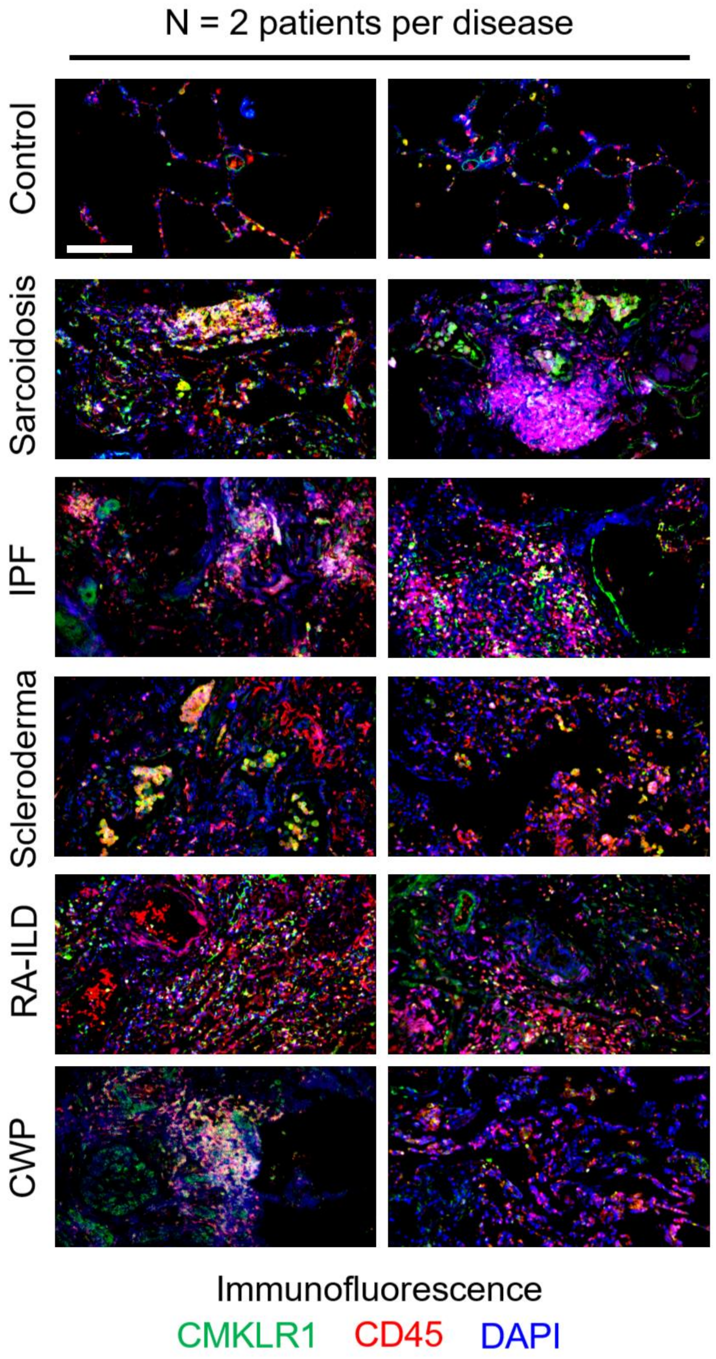

Figure S12. CMKLR1 expression in fibrotic lung diseases. Additional examples of CMKLR1 (green) and CD45 (red) immunostaining in the lungs of controls and patients with fibrotic lung diseases. For each disease, the images in the left-hand and right-hand columns represent different patients. Images from the third patients from each disease category are shown in Fig. 8 in the main text. Abbreviations: IPF = idiopathic pulmonary fibrosis; CWP = coal workers' pneumoconiosis; RA-ILD = rheumatoid arthritis associated interstitial lung disease. Scale bar = 200 µm.

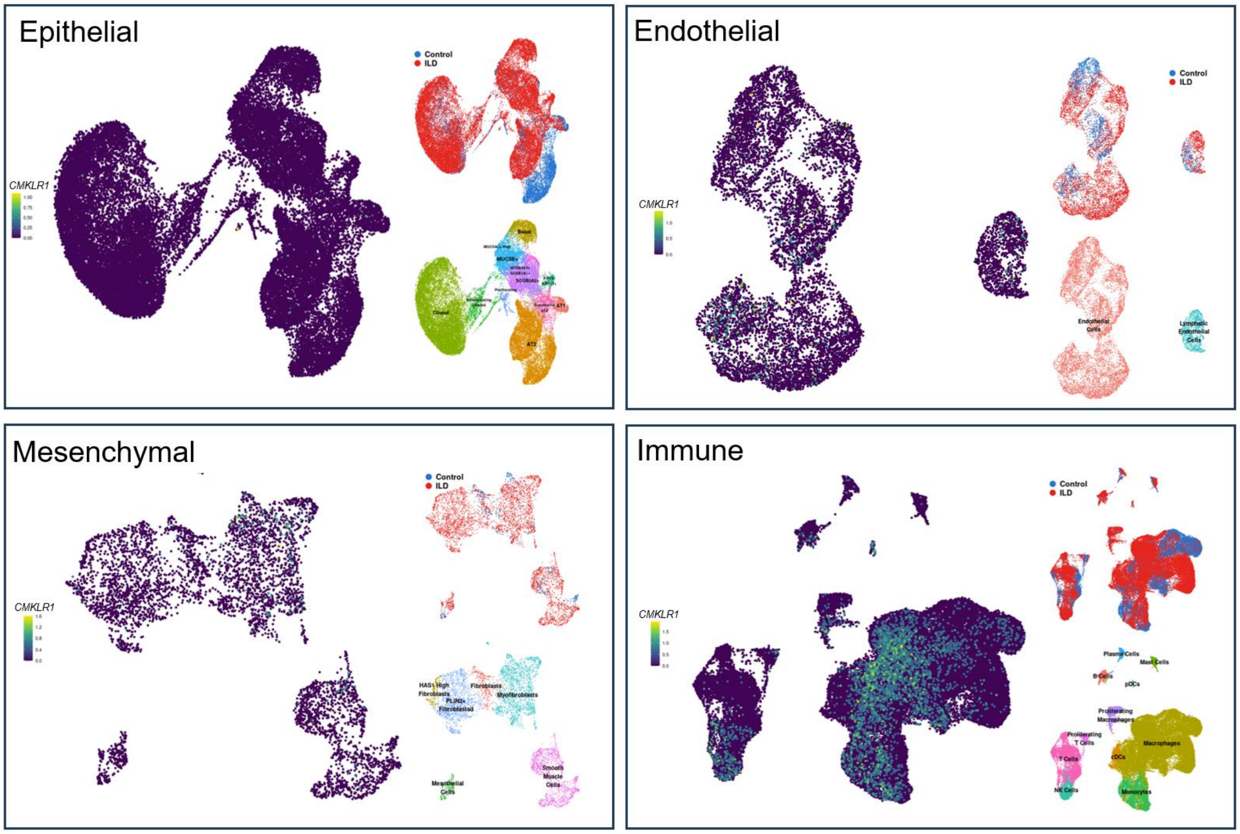

Figure S13. Secondary analysis of *CMKLR1* expression by various immune and non-immune cells of the lungs from control participants and patients with ILD. UMAP representation of *CMKLR1* expression by various cell types using a publicly available dataset (GEO accession: GSE135893). A low level of CMKLR1 expression is present in various lung epithelial (top left), endothelial (top right), and mesenchymal (bottom left) cells, which is noticeably lower than that of immune cells (bottom right), particularly macrophages. UMAP plots were generated using the IPF Cells Atlas platform (http://www.ipfcellatlas.com).

Table S1. Characteristics of controls and patients with fibrotic lung diseases whose lungs were used for histology.

Disease	Age (years)	Gender
Control	53	Male
	53	Male
	51	Female
Idiopathic pulmonary fibrosis	65	Male
	61	Female
	63	Male
Coal worker's pneumoconiosis	58	Male
	65	Male
	54	Male
Rheumatoid arthritis	62	Male
	34	Male
	54	Male
Sarcoidosis	66	Male
	57	Male
	55	Female
Scleroderma	64	Female
	60	Male
	61	Female

Table S2. List of general reagents.

Reagent	Company	Catalog number
Bleomycin	Sigma	B8416-15UN
C57BL/6 plasma	Innovative Research	IGMSC57PLAK2E10ML
Cell strainer (70 um)	VWR	10199-656
Clear 96-well plate	Fisher	FB012931
DNase I	Sigma	D4527-10KU
Fetal bovine serum	Gibco	10082147
Geneticin	Fisher	10-131-035
Ham's F-12K medium	Thermo Fisher	21127022
Hydroxyproline assay kit	Sigma	MAK008-1KT
Hygromycin B	Thermo Fisher	10687010
Lipofectamine 3000	Thermo Fisher	L3000008
Low retention tubes (for radiolabeling)	Eppendorf	022431081
Phosphate-buffered saline (PBS)	Lonza	17-512F
Penicillin/streptomycin	Gibco	15070-063
Poly-D-lysine	Thermo Fisher	A3890401
Qiagen plasmid Maxi kit	Qiagen	12162
RBC lysis buffer (10X)	BioLegend	420301
Rodent intubation stand	Braintree Scientific	RIS 100
RPMI-1640	Gibco	11879-020
Wheat germ agglutinin	Fisher	W32466

Table S3. Plasmids for transfections.

Plasmid	Company	Catalog number
$G_{\alpha 15}$	Sino Biological	HG12687-CF
hCMKLR1	cDNA Resource Center	CMKL100000
mCCRL2	Origene	MG205519
mCMKLR1	Origene	MC208581
mGPR1	Origene	MG205364

Table S4. List of antibodies and reagents used for flow cytometry.

Reagent	Company	Catalog number
Anti-CD11b-PE	BioLegend	101208
Anti-CD11c-PerCP	BioLegend	117326
Anti-CD24-AF700	BioLegend	101836
Anti-CD45-BV421	BioLegend	103134
Anti-CD64-APC	BioLegend	139306
Anti-CMKLR1-AF488	R&D Biosystems	FAB7610G
Anti-Ly6C-APC-Cy7	BioLegend	128026
Anti-Ly6G-BV395	BD Biosciences	563978
Anti-MHC-II-BV605	BioLegend	107639
Anti-SiglecF-BV510	BD Biosciences	740158
DAPI	BD Biosciences	564907
FITC Easy Calibration Kit, 5 peaks	Spherotech	ECFP-F1-5K
IgG_{2B} isotype control-AF488	R&D Biosystems	IC013G
Mouse Fc block	BD Pharmingen	553141
Precision count beads	BioLegend	424902

Table S5. List of antibodies and reagents used for immunostaining.

Antibody	Company	Catalog number
Anti-rabbit IgG (H+L) - Cy5	Jackson ImmunoResearch	711-175-152
Anti-rat IgG (H+L) - Cy3	Jackson ImmunoResearch	712-165-150
ProLong gold antifade mountant with DAPI	Thermo Fisher	P36931
Rabbit anti-mouse CMKLR1	LSBio	B12924
Rat anti-mouse F4/80	Thermo Fisher	14-4801-81
Rabbit anti-human CMKLR1	Abcam	ab306554
Mouse anti-human CD45	Thermo Fisher	Ma1-19111

Other Supplementary Materials:

Supplemental data file S1. A tab-delimited table of gene expression markers for each cell type. The results of an unpaired Wilcoxon rank-sum test of normalized gene expression from cells in each cell type versus all other immune cell types. Genes were only tested if there was a log2 fold change value greater than 0.5. Marker genes were tested independently within each dataset.

Supplemental data file S2. A tab-delimited table representing the percent of cells with detected *CMKLR1* expression and the average normalized expression, for each disease condition, in each cell type, in each dataset. The "3prime" dataset corresponds to the data from Adams *et al*. (20); the "5prime" dataset corresponds to the data from Habermann *et al*. (21).

Supplemental data file S3. Differentially expressed genes between the patients with high *vs.* low expression of *CMKLR1* by BAL cells.

Supplemental data file S4. Tabular representation of the data used in the manuscript figures.

Supplemental data file S5. Tab-delimited table representing the data related to individual cells in the scRNA-seq analysis.
